# ToxAssay: a hierarchical model-driven tool for advanced toxicogenomics biomarker discovery

**DOI:** 10.1093/bioinformatics/btaf561

**Published:** 2025-10-11

**Authors:** Md Masud Rana, Md Nurul Haque Mollah, Mohammed H Albujja, Sibte Syed Hadi, Fan Liu

**Affiliations:** National Genomics Data Center, Beijing Institute of Genomics, Chinese Academy of Sciences and China National Center for Bioinformation, Beijing, 100101, China; University of Chinese Academy of Sciences, Beijing, 100049, China; Bioinformatics Laboratory, Department of Statistics, University of Rajshahi, Rajshahi, 6205, Bangladesh; Department of Forensic Sciences, College of Criminal Justice, Naif Arab University for Security Sciences, Riyadh, Kingdom Saudi Arabia; Department of Forensic Sciences, College of Criminal Justice, Naif Arab University for Security Sciences, Riyadh, Kingdom Saudi Arabia; Department of Forensic Sciences, College of Criminal Justice, Naif Arab University for Security Sciences, Riyadh, Kingdom Saudi Arabia

## Abstract

**Motivation:**

Understanding the genetic basis of drug-induced toxicity is crucial for drug development. In-silico analysis of toxicogenomics datasets facilitates early detection of toxicity biomarkers. However, existing tools struggle with the complex interdependencies among hierarchically structured variables, leading to inaccurate biomarker identification. To address this limitation, we developed a Hierarchical Linear Model (HLM) and implemented it in the R package ToxAssay, offering extensive functionality for comprehensive toxicity assessment.

**Results:**

ToxAssay outperforms existing methods by improving biomarker detection and computational efficiency. Applied to glutathione depletion-induced toxicity, it prioritized 71 key genes and identified 26 core genes with high discriminative accuracy (AUC = 0.97) and strong cross-correlation (Pearson’s *r *= 0.88) with external datasets. Additionally, our advance outcome pathway (AOP) analysis algorithm uncovered disease outcomes linked to glutathione depletion. These findings provide precise insights into the molecular mechanisms driving drug-induced toxicity.

**Availability and implementation:**

ToxAssay is available as an open-source R package at https://github.com/Fun-Gene/toxassay.

## 1 Introduction

Drug-induced organ toxicity damages organs in the body due to the adverse effects of a drug or chemical substance, leading to a high failure rate of new drug candidates in clinical trials. Approximately 30% of drug candidates fail due to unwanted or unmanageable toxicity between 2010 and 2017 (Dowden and Munro 2019; Sun *et al.* 2022). Failing to identify toxicity during the testing stage can risk public health with delayed adverse effects. Early detection of toxicity biomarkers can reduce costs, rejection rates, and the need for human and animal testing, enhancing patient safety (Khan *et al.* 2014). In-silico analysis of toxicogenomics data has proven to be an effective and powerful approach for the early detection of toxicity biomarkers (Pognan *et al.* 2023).

Several toxicogenomics databases have been established, among which Open TG-GATEs (Igarashi *et al.* 2015), DrugMatrix (Ganter *et al.* 2006) and Comparative Toxicogenomics Database (CTD) (Davis *et al.* 2023) are the three main public databases directly linking toxicity with gene expression data (Alexander-Dann *et al.* 2018). The Open TG-GATEs, designed for toxicogenomics, emphasizes toxic doses and includes compounds known for toxic effects. DrugMatrix, on the other hand, covers a wider range of chemicals, focusing on effective doses. The CTD stands out for its comprehensive curation of scientific literature, linking environmental factors to human health with a vast repository of chemicals, genes, and diseases. These databases have enabled extensive investigations, such as generating biomarker signatures and characterizing mechanisms of toxicological endpoints in the liver, including glutathione depletion, phospholipidosis, cholestasis, fibrosis, cirrhosis, and carcinogenesis. Pharmaceutical research nowadays heavily relies on these findings in understanding the drug toxicity (Uehara *et al.* 2010; Schomaker *et al.* 2019).

Commonly used open-source software tools for the analysis of large-scale toxicogenomics datasets include ToxicoDB (Nair *et al.* 2020) and Toxygates (Nyström-Persson *et al.* 2013, 2017). ToxicoDB provides synchronized compound annotations, dynamic compound plots based on time and dosage, and pathway analysis related to toxicity. Toxygates offers an integrated platform for ranking compounds using pattern-based approaches reliant on relevant candidate gene expression values. The core methods used for identifying differentially expressed genes (DEGs) in ToxicoDB, and Toxygates are limma and Welch’s *t*-test, respectively. Moreover, a majority of independent studies in toxicogenomics used Welch’s *t*-test (or ANOVA) and limma for identifying DEGs (Kiyosawa *et al.* 2004; Kiyosawa *et al.* 2007; Ellinger-Ziegelbauer *et al.* 2008; Uehara *et al.* 2008; Gao *et al.* 2010; Huang and Tung 2017).

Although these existing tools have demonstrated their applicability from different perspectives, there is significant room for improvement. Firstly, different methods often produce significantly divergent *P*-values on same datasets due to a lack of consideration for the hierarchical structure of experimental design (Kulkarni *et al.* 2022). For example, ToxicoDB is designed to analyze single-compound data, accounting for dose and time factors in the design matrix to identify DEGs, but it does not consider the interdependencies between time and dose across a group of similar compounds. In contrast, Toxygates follows a standard procedure to identify DEGs by comparing two sets of compounds (e.g. toxicity-positive and toxicity-negative), but it simplifies the approach by pooling samples into groups, overlooking the specific interdependencies of dose and time factors. Secondly, there has been limited methods for AOP analysis that connect molecular-level biological perturbations to adverse outcomes. Lastly, user-friendly tools for automated post-marker discovery analysis are lacking, making these tasks challenging for wet-lab researchers. These limitations underscore the necessity of developing a sophisticated tool capable of analyzing various toxicities, selecting relevant biomarkers with reduced redundancy, and addressing a broad spectrum of inquiries with optimized statistical power.

In this study, we present a novel statistic for identifying DEGs using HLM that takes into account the hierarchical data structure inherent in toxicogenomics data. We demonstrate the statistical power of our model through extensive computer simulations. We also developed an association rule mining algorithm for AOP analysis that maps compound perturbation data to disease data from CTD. We implemented our newly developed statistical models and algorithms in a user-friendly R package ToxAssay, offering extensive functionality for post-marker discovery analysis. To demonstrate its utility, we applied ToxAssay to analyze glutathione depletion-induced toxicity, a key endpoint, using the Open TG-GATEs dataset.

## 2 Results

### 2.1 Overview of ToxAssay

**Figure 1.**
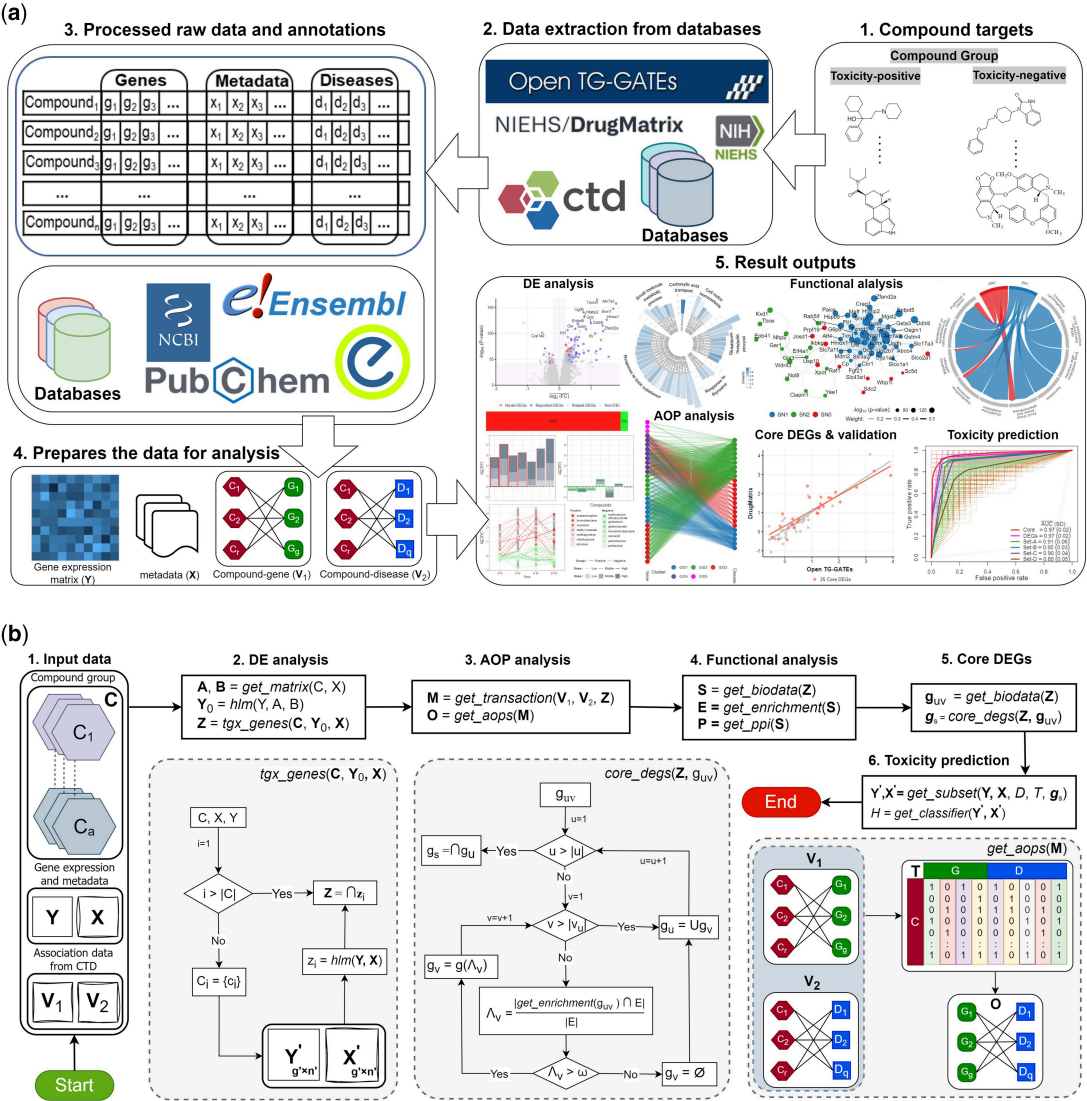
Overview of ToxAssay. (a) workflow of ToxAssay in five steps. Step 1: users specify a list of compounds (e.g. toxicity-positive and toxicity-negative) for comparison to study targeted toxicity. Step 2: extracts raw data from existing databases. Step 3: The raw data is annotated with compound and gene identifiers. Step 4: formats and prepares the data for analysis. Step 5: ToxAssay provides multiple analysis functions, generating tables and graphical outputs for DEGs, gene expression patterns, AOP associations, functional pathways, PPI networks, and toxicant prediction. Note that Steps 2–4 can be skipped if the input data (**Y**, **X**, **V_1_**, and **V_2_**) is already available. (b) Algorithm flowchart highlighting main functions in ToxAssay. A brief description of the flowchart is provided in Supplementary Note. Key functions *tgx_genes*, *core_degs*, and *get_aop* are detailed in the gray dotted boxes at the bottom.

The workflow of ToxAssay is depicted in Fig. 1 and the key functions of ToxAssay are described in Supplementary Method A. In brief, ToxAssay is an R-based software package designed for the comprehensive evaluation of drug-induced toxicity utilizing complex toxicogenomics databases, including perturbation data from sources such as Open TG-GATEs and DrugMatrix, along with manually curated relational data from the CTD. The package offers a comprehensive suite of functions, encompassing: (i) identification of molecular markers for targeted toxicity, including DEGs, advance outcome pathways (AOPs), functional pathways, and Protein–Protein Interaction (PPI) networks; (ii) development of optimized machine learning classifiers for predicting the targeted toxicity in test samples of compounds.

An important innovative component of ToxAssay is that it implements a newly developed HLM that model gene expressions, considering factors such as compounds, dosages, and time points, in a nested data structure (compound → dose → time point) (Supplementary Fig. S1). Details regarding the development of HLM are provided in Methods. In brief, let $y_{\mathrm{jklm}}\;$represent the gene expression value measured in the *m*-th replication at the *l*-th time point following exposure to the *k*-th dose of the *j*-th compound. For each compound-dose-time combinations, the HLM is written as $y_{\mathrm{jklm}}=\lambda_{\mathrm{jkl}}+\varepsilon_{\mathrm{jklm}}$, where $\varepsilon_{\mathrm{jklm}}∼N(0,\;\sigma_{y}^{2})$ denotes the residual error term and $\lambda_{\mathrm{jkl}}∼N(\gamma_{jk},\;\sigma_{\lambda }^{2})$ represents the intercept for time level. The intercept for the dose level is $\gamma_{jk}∼N(\beta_{j},\;\sigma_{\gamma }^{2})$, where $\beta_{j}$ is the intercept at the compound level.

Let $C_{1},C_{2},\ldots ,C_{a}$ be a set of compound groups (typically a=2, representing targeted toxicity-positive and toxicity-negative) to be compared for assessing targeted toxicity, where each $C_{i}$ contains $b_{i}$ compounds with similar toxicity characteristics. Then, the intercept of compounds in the *i*-th group is $\beta_{j(i)}∼N(\mu +\tau_{i},\;\sigma_{\beta }^{2})$, where μ is the overall intercept (ie, mean) across all compounds and $\tau_{i}$ is the mean effect of the *i*-th compound group. The null hypothesis of $H_{0}:\tau_{1}=\tau_{2}\cdots =\tau_{a}=0$ is tested using a *F*-statistic with (a−1) and (n-p) degrees of freedom, adjusted for the extent of dependency in the data, which can be captured by the overall intracluster correlation (ICC), $\rho =\sigma_{o}^{2}/(\sigma_{o}^{2}+\sigma_{\varepsilon }^{2})$, where $\sigma_{o}^{2}=\sigma_{\beta }^{2}+\sigma_{\gamma }^{2}+\sigma_{\lambda }^{2}$ is the total of the intercept variances. Here, p and n represent the total number of possible clusters and samples in the data, respectively.

Let ${DE}_{0}$ be the initial set of DEGs identified by the proposed *F*-statistic. To refine the initial set and remove genes influenced by chemical-specific outlying expressions, ToxAssay generates gene sets, ${DE}_{j}(j=1,2,\ldots ,r=\sum_{i=1}^{a}b_{i})$ using a cross-validation framework. Finally, the reduced DEGs set is obtained as: $\mathrm{DEGs}=\overset{r}{\underset{j=1}{\cap }}{DE}_{j}$ (see Methods for details).

ToxAssay further utilizes DEGs for AOPs analysis using a proposed algorithm developed by association rule mining. The DEGs is subsequently used for functional pathway and PPI analysis to elucidate underlying molecular mechanisms and identify most interconnected and biologically relevant core DEGs (CDEGs) (see Methods for details). Finally, the identified gene signature is utilized to develop machine learning classifiers, aimed at predicting the targeted toxicity of the toxicant to be tested.

### 2.2 Power of ToxAssay via computer simulations

Computer simulations were carried out to assess the performance of ToxAssay in terms of statistical power (scenario 1) and type-I error (α) (scenario 2), in comparison with two other competing software packages, ToxicoDB (Nair *et al.* 2020) and Toxygates (Nyström-Persson *et al.* 2013; 2017). The simulations were conducted under both balanced (equal number of compounds in the targeted toxicity-positive and toxicity-negative groups) and unbalanced (unequal number of compounds) designs.

**Figure 2.**
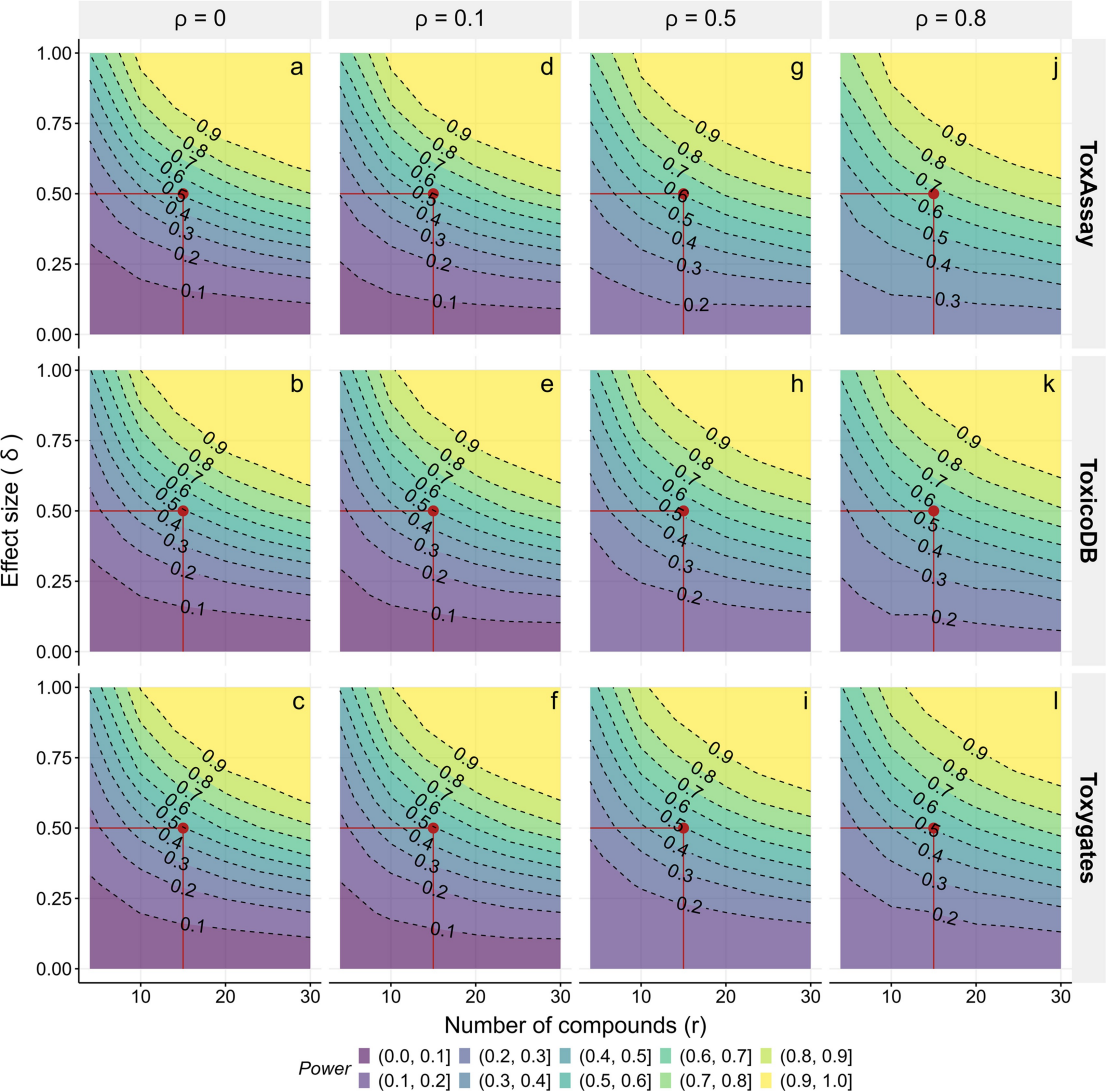
Power comparison between ToxAssay, ToxicoDB and Toxygates via simulations under balanced design. Gene expression data were simulated for 10 000 genes, with 10% exhibiting a true effect, according to the parameter grid: δ∈{0, 0.1, 0.2,…,1}, ρ∈{0, 0.1, 0.5, 0.8} and r∈{4, 6, 10, 14, 20, 30}. The power of ToxAssay, ToxicoDB, and Toxygates was evaluated under four conditions: ρ=0 (a–c), ρ=0.1 (d–f), ρ=0.5 (g–i), and ρ=0.8 (j–l). Each scenario was assessed using 100 replicates at α=0.05. The statistical power of each testing framework is illustrated using LOESS smoothing. Contour lines indicate combinations of r and δ that yield equivalent statistical power, quantitatively expressed. A color gradient highlights regions of varying statistical power, with areas of low power shown in purple and areas of high power in yellow. The x-axis represents the number of compounds (r), and the y-axis represents the effect size (δ), while ρ denotes the level of data dependency. Notably, ToxAssay demonstrated increased power as ρ increased.

Scenario 1 compares the statistical power between ToxAssay, ToxicoDB, and Toxygates in detecting DEGs across various combinations of effect sizes (δ) and compound numbers (r) under varying levels of overall ICC (ρ). In a balanced design with no data dependency (ρ=0), all packages exhibit equivalent power across all combinations of δ and r (Fig. 2a–c). However, as ρ increases, ToxAssay consistently outperforms the other packages, demonstrating superior power in every combination of δ and r. Specifically, at low dependency (ρ=0.1), power increases by approximately 5% (Fig. 2d–f). At moderate dependency (ρ=0.5), power improves by roughly 10% (Fig. 2g–i), and at high dependency (ρ  =  0.8), power surges by approximately 20% when r is 15 and δ is 0.50 (Fig. 2j–l). A similar trend is observed under unbalanced designs with the same parameter settings (Supplementary Fig. S2).

Scenario 2 explores the type-I error rate (α) across different r values under both balanced and unbalanced design (Supplementary Fig. S3). Simulation results indicate that α consistently remains at 5% or slightly above, under varying r. For perfectly non-DEGs, where there is no variation in the intercepts, α consistently stabilizes at 5%. Even in the presence of small variation in the intercepts, allowing for minor biological noise, there is a slight elevation in α, regardless of changes in r. This underscores the high stability of α in the ToxAssay.

### 2.3 ToxAssay R package and computational efficiency

ToxAssay is a user-friendly, open-source R package available at https://github.com/Fun-Gene/toxassay. Toxicogenomics data analysis can become computationally intensive, especially with large datasets and repetitive tasks across multiple endpoints. We assessed the computational efficiency of ToxAssay with ToxicoDB and Toxygates, using simulated data (1, 5, 10, 20, and 50 thousand genes; 6, 10, 20, 30, 40, and 50 compounds). ToxAssay consistently outperformed all benchmarks in terms of peak memory usage and computation time in all dataset configurations (Supplementary Fig. S4).

### 2.4 Identification of DEGs associated with glutathione depletion

To empirically showcase ToxAssay’s power and proficiency in evaluating toxicological endpoints, we applied it to analyze the Open TG-GATEs dataset for glutathione depletion-induced toxicity, comparing our results with four previous studies. These previous studies proposed different and partially overlapping gene sets as biomarkers for early detection of glutathione depletion, ie 53 (53 probes, set-A) (Kiyosawa *et al.* 2004), 145 (161 probes, set-B) (Kiyosawa *et al.* 2007), 64 (78 probes, set-C) (Gao *et al.* 2010), and 385 (460 probes, set-D) (Yamauchi *et al.* 2011) (Supplementary Table S1). Of note, there was a remarkable scarcity of overlapping genes between any two previously proposed gene sets (ranging from 2.1% between set-A and set-B to 35.9% between set-C and set-D).

**Figure 3.**
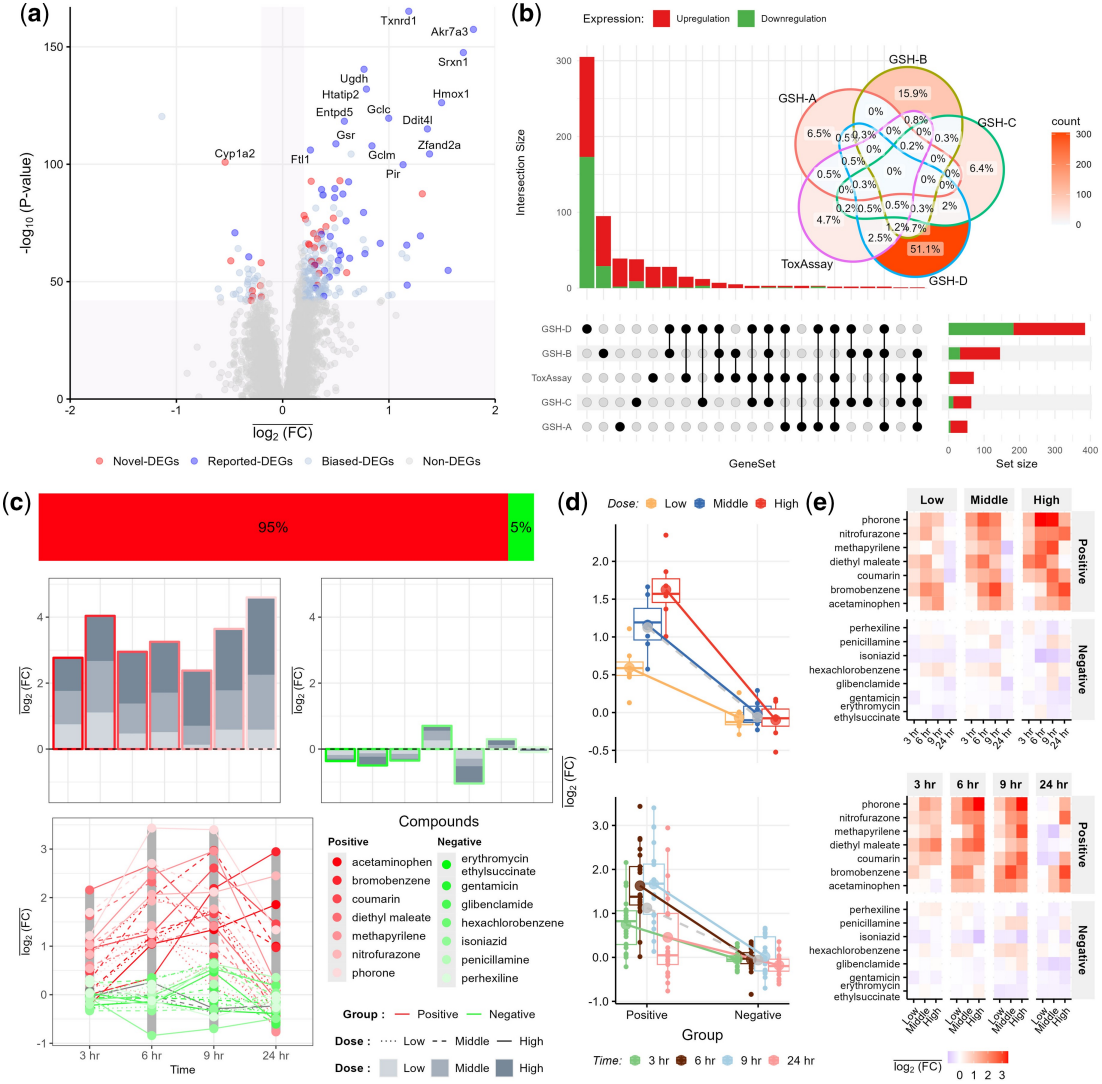
DEG identification in glutathione depletion. (a) Volcano Plot of DEGs. The x-axis represents the average log_2_(FC) in gene expression between GDPCs and GDNCs across dose and time, while the y-axis shows the −log_10_  *P*-values. Red dots indicate novel DEGs, blue dots indicate previously reported DEGs, light blue dots represent chemical-specific (biased) DEGs, and grey dots denote non-DEGs. (b) Venn diagram and upset plot of biomarker genes. The overlap and uniqueness of gene sets identified by ToxAssay and four prior studies. The ellipses represent intersecting gene sets for ToxAssay (purple), Set-A (red), Set-B (olive), Set-C (green), and Set-D (blue). The bar plot displays the count of unique upregulated and downregulated genes. (c) The GDPCs regulated approximately 95% of the overall expression of top DEG *Txnrd1*, in contrast to the minimal expression regulated by the GDNCs. *Txnrd1* exhibited higher average log_2_(FC) in the GDPCs group across compounds, doses, and time points compared to GDNCs. (d) The figure illustrates clustering in gene expression levels using *Txnrd1* as an example. Compared to the traditional *t*-test (gray dashed line), HLM provides a significantly better fit across doses (top) and time points (bottom). (e) Heatmap of *Txnrd1* expression clearly distinguishes GDPCs and GDNCs groups at different doses (top) and time points (bottom).

Out of the 15 027 unique genes in the gene pool derived from > 31 000 GeneChip^®^ RAE 230A gene probes, ToxAssay identified 71 significant DEGs (P<0.05 after Bonferroni correction, Fig. 3a, Supplementary Table S2). The overall ICC values of these genes in TG-GATEs were indeed very high (mean = 0.83, sd = 0.06, Supplementary Fig. S5), confirming a high degree of data dependency and consistent with our simulation analysis. Among these 71 genes, 60.6% (43/71) overlapped with previously reported gene sets, with increased pair-wise overlaps with any previously proposed gene set (12.7% with set-A, 22.5% with set-B, 14.1% with set-C, and 46.5% with set-D, Fig. 3b). We confirm the overlapping finding of *Txnrd1* in set-B and set-D as the most significantly DEG in our study. The average fold change expression ($\overset{-}{\log_{2}\left(\mathrm{FC}\right)}$) of *Txnrd1* across different levels between glutathione-depletion positive compounds (GDPCs) and glutathione-depletion negative compounds (GDNCs) groups clearly demonstrates significant difference, as well as cluster-related variations and patterns in the data (Fig. 3c–e).

There was strong evidence supporting our newly identified genes (28/71) being functionally involved in glutathione depletion. For example, *Mgst2* and *G6pd* have been long used as the index genes for ranking GDPCs but failed to be identified by all previous studies (Nyström-Persson *et al.* 2013). We also observed significant differential expression and cluster-related variations in these genes across different levels (Supplementary Fig. S6). In addition, *Txn1* and *Raf1* have been reported as important contributors to the process of glutathionylation, and others including *Cyp1a2, Maff, Usp10, Zfp703*, *Cp*, *Eaf1*, and *Eif4a1* are known to play significant roles in oxidative stress (Poulsen *et al.* 1998; Lu 2013; Aoiadni *et al.* 2021; Turner and Turner 2021; Sango *et al.* 2022). These lines of functional evidence empirically support the improved statistical power of our method in detecting DEGs.

### 2.5 ToxAssay DEGs unveil AOPs in glutathione depletion

**Figure 4.**
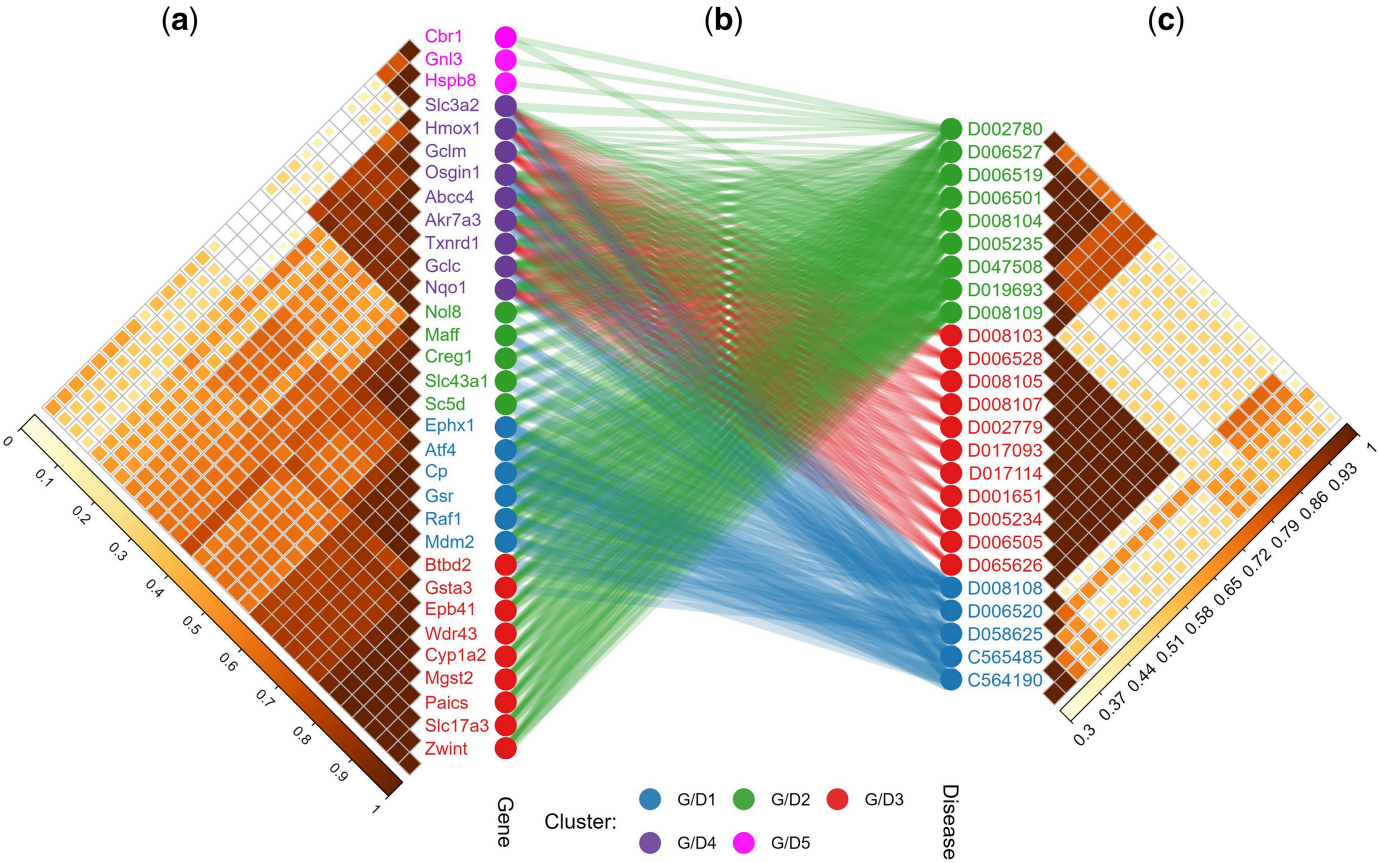
Association between clusters of DEGs and AOPs. (a) DEG clusters. (b) Associations between DEGs and disease MeSH IDs, with the strength of association weighted based on lift values. (c) Disease clusters.

We further explored AOPs related to glutathione depletion, focusing on liver disease outcomes categorized by Medical Subject Headings (MeSH). Through association rule mining analysis, we identified 25 liver diseases linked to 32 DEGs, resulting in 392 significant gene-disease associations (Fig. 4). Consistent with existing knowledge, several AOPs associated with glutathione depletion were highlighted, including non-alcoholic and alcoholic fatty liver disease (MeSH: *D005234*, *D005235*, and *D065626*), cholestasis and cholestatic injury (*D002779*, *D001651*, and *D002780*), hepatitis (*D006505*, *D006519*, *D006520*, and *D019693*), liver cirrhosis (*D008103*, *D008104*, and *D008105*), and chemical/drug-induced liver injury (*D056486* and *D056487*) (Yuan and Kaplowitz 2009; Lu 2020; Vairetti *et al.* 2021). A more comprehensive list of AOPs induced by glutathione depletion and their associated genes is provided in Supplementary Table S3.

Cluster analysis of gene-disease associations identified five distinct gene clusters (G1 to G5), each showing similar association patterns within the cluster but differing between clusters, suggesting shared molecular mechanisms within each group (Fig. 4a and c). The six genes in G1 (*Ephx1*, *Atf4*, *Cp*, *Gsr*, *Raf1*, and *Mdm2*) are mainly involved in cellular stress responses and apoptosis regulation. The five genes in G2 (*Nol8*, *Maff*, *Creg1*, *Slc43a1*, and *Sc5d*) contribute to cellular homeostasis and metabolic regulation. The nine genes in G3 (*Btbd2*, *Gsta3*, *Epb41*, *Wdr43*, *Cyp1a2*, *Mgst2*, *Paics*, *Slc17a3*, and *Zwint*) function in detoxification and cellular metabolism. The nine genes in G4 (*Txnrd1*, *Nqo1*, *Hmox1*, *Gclm*, *Gclc*, *Akr7a3*, *Abcc4*, *Osgin1*, and *Slc3a2*) play key roles in protecting against oxidative stress, likely regulated by nuclear factor erythroid 2–related factor 2 (Nrf2). Finally, the three genes in G6 (*Cbr1*, *Gnl3*, and *Hspb8*) are associated with cellular protection and stress response. While these annotations align with existing literature, further statistical validation is required to confirm their significance in this specific dataset.

Similarly, cluster analysis revealed three distinct disease clusters (D1 to D3). Diseases in D1 were predominantly explained by genes from G1, G2, and G4, indicating a shared molecular basis between these disease and gene clusters. D2 was influenced by a wide spread of the tested genes, making it difficult to attribute specific functional annotations to this cluster. In contrast, D3 exhibited a more specific gene-disease relationship, with genes from G4 being exclusively associated with diseases in D3, including fatty liver (*D005234* and *D065626*), cholestasis (*D002779* and *D001651*), liver cirrhosis (*D008103* and *D008105*), hepatitis (*D006505*), hepatocellular carcinoma (*D006528*), liver failure (*D017093* and *D017114*), and drug-induced liver injury (*D006528*). These findings overall reveal distinct gene and disease clusters associated with glutathione depletion, offering insight into potential molecular mechanisms.

### 2.6 Core differentially expressed genes (CDEGs) in glutathione depletion

**Figure 5.**
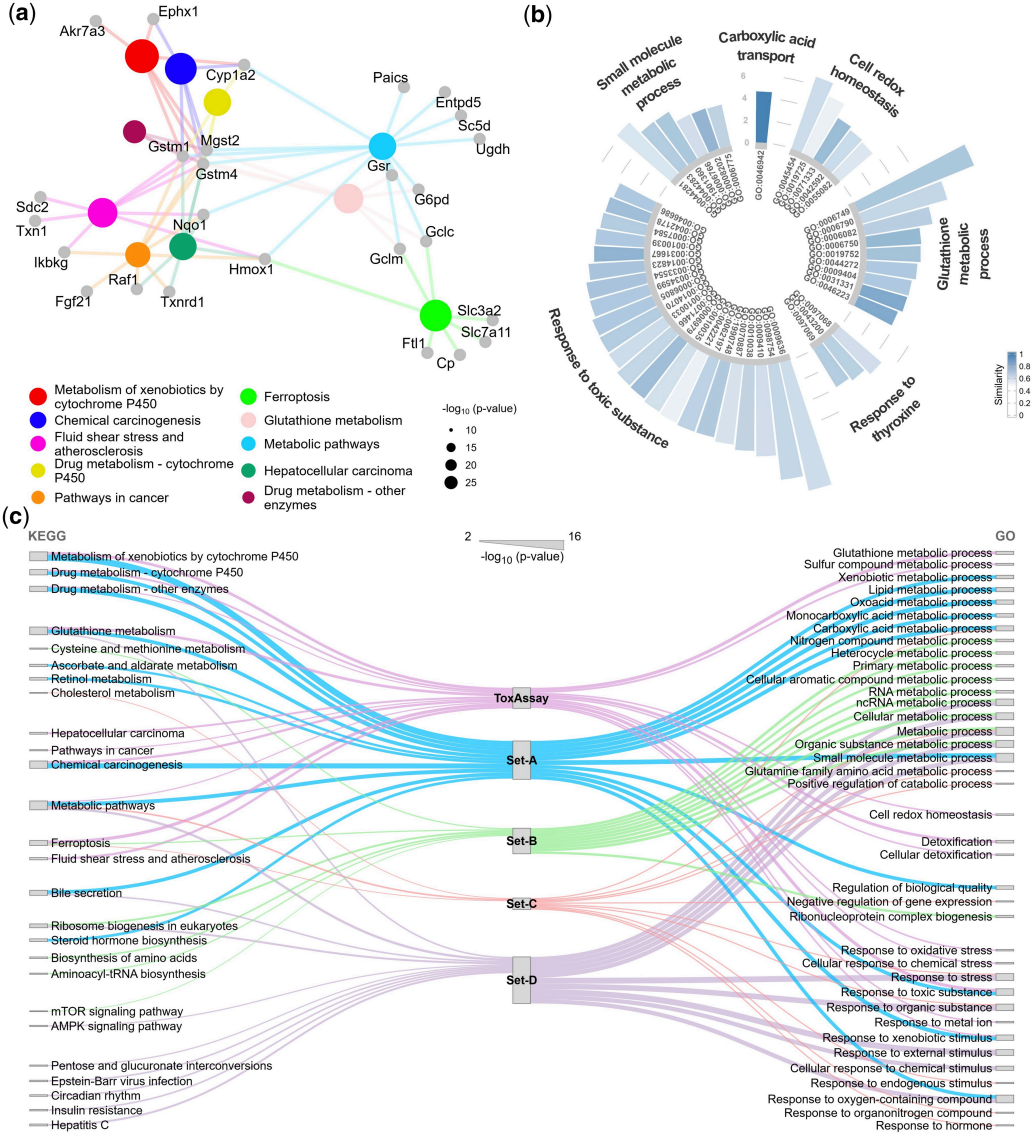
Enriched pathways in glutathione depletion. (a) KEGG pathway enrichment. A total of 26 DEGs were enriched in the top 10 KEGG pathways (Q < 0.01). The size of the nodes representing pathway terms corresponds to the −log_10_  *P*-values of enrichment. (b) GO biological process clusters. From 46 enriched GO biological process terms, six parent clusters were identified using a high similarity threshold of 0.9. The bars within each parent pathway term represent the enriched terms, with the intensity of blue indicating the similarity within clusters. The x-axis displays the GO IDs, and the y-axis shows the −log_10_  *P*-values of the enriched terms. (c) Comparative Enrichment: KEGG and GO terms enriched by ToxAssay were more relevant to glutathione depletion than those identified in previous studies. The thickness of the lines reflects the −log_10_  *P*-values of the pathway terms.

We explored the functional roles of 71 significant DEGs using databases KEGG (Kanehisa *et al.* 2017), gene ontology (GO) (Harris *et al.* 2004), Reactome (Croft *et al.* 2011) and Wikipathway (Slenter *et al.* 2018) (Supplementary Table S4). Notably, we identified significant enrichment in several key metabolism-related pathways, including metabolism of xenobiotics by cytochrome P450 ($p=1.47\times 10^{-10}$), glutathione metabolism ($p=2.51\times 10^{-7}$) and metabolic pathways ($p=4.66\times 10^{-7}$), as well as in various cancer-related pathways such as ferroptosis ($p=2.25\times 10^{-8}$), chemical carcinogenesis ($p=2.25\times 10^{-8}$) and hepatocellular carcinoma ($p=6.08\times 10^{-5}$) in the KEGG database (Fig. 5a). Cluster analysis based on the semantic similarity of GO biological process terms highlighted clusters related to cellular redox homeostasis, response to toxic substances, response to thyroxine, as well as glutathione metabolism and small molecule metabolic processes (Fig. 5b). Our findings suggest that our primary set of 71 DEGs is significantly more relevant to glutathione depletion than previously identified gene sets (Fig. 5c), a finding further supported by pathway analysis using Reactome and Wikipathways databases (Supplementary Fig. S7).

**Figure 6.**
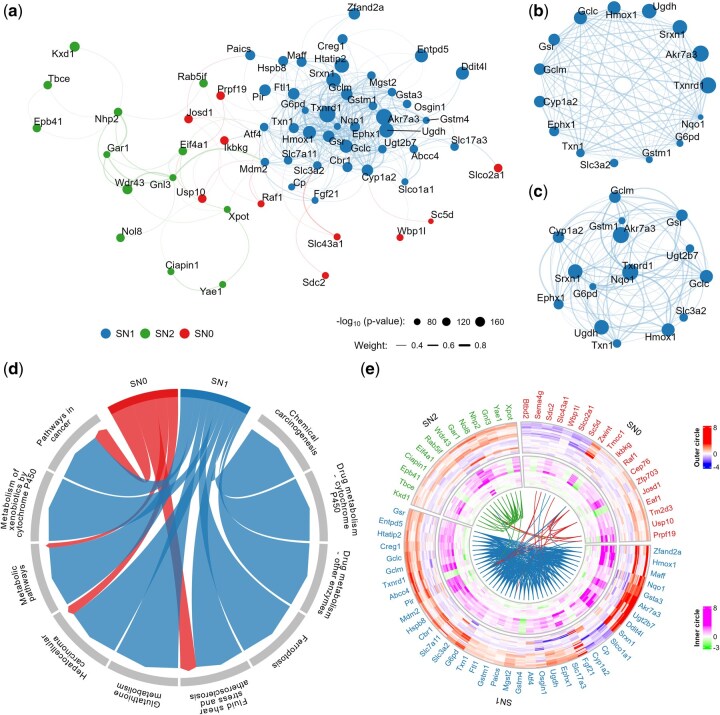
PPI subnetworks and hub genes functionally involved in glutathione depletion. (a) The PPI network of DEGs revealed three distinct subnetworks, with Subnetwork 1 (SN1) being the densest, containing all hub genes identified by the degree-based centrality measure (b) or the eigenvector-based centrality measure (c). (d) SN1 showed the most widespread enrichments in pathways identified in KEGG. (e) Expression patterns of genes in SN1 clearly differentiate samples from GDPCs and GDNCs. The inner circle heatmap is generated from average log_2_(FC) data at 24-hour high dose, while the outer circle heatmap is based on average log_2_(FC) data across all time points at high dose levels.

A PPI analysis of the 71 DEGs using STRING (Szklarczyk *et al.* 2021) resulted in a dense PPI network consisting of 70 nodes and 355 edges. Except one, all the 71 DEGs are protein-coding, and among which, 62 genes formed an interconnected network within the PPI framework (Fig. 6a, Supplementary Table S5). By applying a degree centrality threshold of 20 within the dense PPI network, we delineated a core network of 15 hub genes (Fig. 6b). Additionally, a parallel analysis using an eigenvalue centrality threshold of 0.7 confirmed a similar set of hub genes, along with *Ugt2b7* (Fig. 6c).

In order to minimize the variability in results from differing algorithmic choices, we employed three widely used algorithms including edge-betweenness centrality (EBC) (Girvan and Newman 2002), Walktrap (Clauset *et al.* 2004), and Fastgreedy (Pons and Latapy 2005) to identify co-regulated subnetworks within the PPI network. Genes in the subnetworks identified by the EBC algorithm were found to play a dominant role in identified KEGG pathways and displayed distinctly clear clustering patterns in samples (Fig. 6d and e). Similarly, genes in the key subnetworks identified by the Walktrap and Fastgreedy algorithms demonstrated a significant influence on identified KEGG pathways and exhibited distinct clustering patterns (Supplementary Fig. S8). Genes in the key subnetworks identified by all three algorithms, which play a dominant role in KEGG pathways, are largely common and also exhibit a similar dominant effect in other pathway databases (Supplementary Fig. S9).

Using a soft threshold of ω≥0.25 in our core DEGs identification method (see Methods), we identified 26 CDEGs (*Txnrd1*, *Akr7a3*, *Srxn1*, *Ugdh*, *Hmox1*, *Gclc*, *Gsr*, *Gclm*, *Ftl1*, *Zfand2a*, *Cyp1a2*, *Hspb8*, *Ephx1*, *Txn1*, *Slc3a2*, *Mgst2*, *Gsta3*, *Atf4*, *Slc7a11*, *Slc17a3*, *Gstm1*, *Abcc4*, *Gstm4*, *G6pd*, *Nqo1*, and *Cp*), encompassing all hub genes identified in the PPI network and those strongly associated with significant AOPs (Supplementary Fig. S10). This highly interconnected and biologically significant gene signature underscores their critical roles in glutathione depletion.

### 2.7 Core gene set accurately predicts glutathione-depleting hepatotoxicants

**Figure 7.**
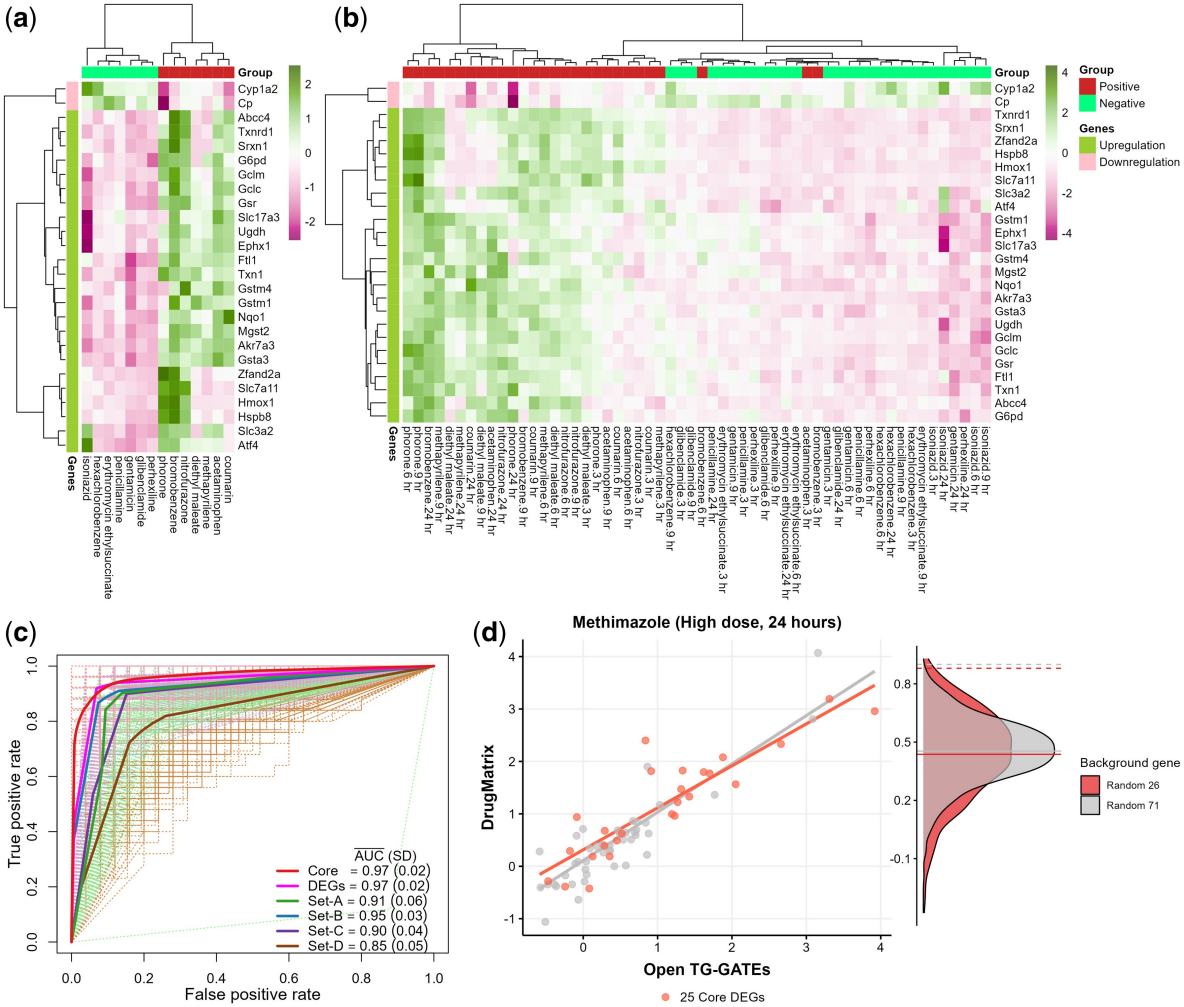
Accuracy in predicting compound groups (GDPCs and GDNCs) using all 71 DEGs or 26 CDEGs. (a) Hierarchical clustering of the 26 CDEGs based on average log_2_(FC) clearly distinguishes samples from high-dose GDPCs and GDNCs groups at the 24-hour time point. (b) Clustering across all time points similarly separates GDPCs and GDNCs. (c) Prediction accuracy of GDPCs and GDNCs using a logistic regression model shows that both the 71 DEGs (pink) and 26 core DEGs (red) perform similarly (AUC = 0.97), outperforming previously proposed gene sets. (d) Cross-correlation analysis of DEG expression between the Open TG-GATEs dataset (used in this study) and the DrugMatrix database (external dataset). DEGs show high cross-correlation, while a randomly drawn set of 71 background genes (gray density) and 26 background genes (red density) from the genome exhibit low correlation. The solid lines on the density plot represent average cross-correlation for the random 26 core DEGs (red) and 71 background genes (gray), with dashed lines representing 26 core genes (red) and 71 DEGs (gray).

Our analysis further probed the predictive capability of the 26-core gene signature on samples subjected to glutathione-depleting agents through both unsupervised hierarchical clustering and supervised classification methods. The unsupervised hierarchical clustering delineated two distinct expression profiles within different data subsets (Supplementary Fig. S11), accurately differentiate samples from GDPCs and GDNCs groups (Fig. 7a and b). This performance of our 26 CDEGs markedly exceeded previously suggested gene sets under the same analyses (Supplementary Fig. S12).

In supervised classification, employing a logistic regression model across 100 iterations within a 10-fold cross-validation framework, we attained notable predictive accuracy (AUC = 0.97), equating to the accuracy obtained using the complete set of 71 DEGs (AUC = 0.97). This significantly surpassed the outcomes of prior studies, which reported an average AUC of 0.86 with a maximum value achieved at 0.95 (Fig. 7c, Supplementary Table S6).

### 2.8 Core gene set shows high cross-correlation across databases

To assess the consistency and robustness of ToxAssay gene signature across independent toxicogenomic platforms, we performed a cross-correlation analysis between the Open TG-GATEs and DrugMatrix datasets, specifically focusing on Methimazole, the only compound common to both datasets with identical experimental setups. Methimazole is a known glutathione-depleting hepatotoxicant, making it an ideal compound for evaluating the generalizability of the ToxAssay gene signature in predicting glutathione depletion-induced hepatotoxicity.

The analysis revealed a strong correlation in gene expression perturbations between the two datasets, with Pearson correlation coefficients of 0.90 for the 71 DEGs and 0.88 for the 26 CDEGs (Fig. 7d). These high correlation values demonstrate the robustness and consistency of the ToxAssay gene signature across distinct experimental platforms, supporting its ability to predict glutathione depletion-related toxicity. For further validation, we compared the observed correlations with those obtained from number-matched random gene signatures. After 10 000 replicates, the random gene signatures yielded much lower correlation values of 0.45 for the 71 gene set and 0.43 for the 26 gene set (*P* < 0.0001). This clear difference between the observed and random correlations highlights the reliability and specificity of the ToxAssay gene signature, confirming its robustness in identifying biomarkers and predicting drug-induced toxicity across diverse experimental contexts.

## 3 Discussion

We present ToxAssay, an R-based software for comprehensive drug-induced toxicity evaluation, utilizing major toxicogenomics databases such as Open TG-GATEs (Igarashi *et al.* 2015), DrugMatrix (Ganter *et al.* 2006), and CTD (Davis *et al.* 2023). While the current implementation focuses on gene expression data from Open TG-GATEs and DrugMatrix, ToxAssay can easily adapt to other datasets like CMap or L1000 (Subramanian *et al.* 2017) and other omics data types, provided the input is properly formatted. Although several databases catalog the toxicity profiles of compounds, they lack molecular-level insights into toxicity mechanisms (Chen *et al.* 2016; Lea *et al.* 2017; Wu *et al.* 2023). ToxAssay addresses this gap by providing functionalities for in-depth molecular analysis and advanced machine learning models to evaluate a wide range of toxicity endpoints.

ToxAssay’s novel HLM enhances the detection of DEGs, outperforming existing tools like ToxicoDB (Nair *et al.* 2020) and Toxygates (Nyström-Persson *et al.* 2013, 2017) in both power and efficiency. Our HLM accounts for the inherent hierarchical structure in the data, such as compound, dose, and time points (compound → dose → time point), which is often overlooked by existing methods. The model is particularly effective when the overall ICC is greater than zero (ie, ρ>0), indicating clustering at any level of the data (compound, dose, or time points). This is evident from both computer simulations and empirical application to glutathione depletion analysis, where our identified gene sets showed greater commonality, functional relevance, and higher discriminative power compared to previously reported sets.

A novel element along with the HLM in ToxAssay is our association rule mining algorithm for AOP analysis. This algorithm improves the mapping of compound perturbation data to disease datasets from curated databases, facilitating the identification of association patterns between clusters of diseases and DEGs. Unlike existing tools such as ToxicoDB (Nair *et al.* 2020) and Toxygates (Nyström-Persson *et al.* 2013, 2017), which lack this functionality, our approach is crucial for linking biological processes to adverse outcomes. Applying this algorithm, we found that the DEG set in cluster G4 is uniquely associated with diseases in cluster D3, revealing a strong connection between oxidative stress genes and liver diseases, a finding consistent with previous mice gene-editing studies that demonstrated the pivotal role of oxidative stress regulation in the progression of liver pathology (Rius *et al.* 2003; Krejsa *et al.* 2010; Ahmed *et al.* 2011; Brennan *et al.* 2017; Lee *et al.* 2019; Canesin *et al.* 2022; Yan *et al.* 2023).

Unlike traditional AOP approaches that emphasize curated pathway analyses, network modeling, or database integration (Fay *et al.* 2017; Spinu– *et al.* 2019; Saarimäki *et al.* 2023; Wiklund *et al.* 2023), our method uniquely incorporates association rule mining to uncover mechanistic links between gene-expression changes and disease states. To our knowledge, this integration has not been benchmarked against existing AOP methods, as no comparable frameworks explicitly apply association rule mining. Thus, our algorithm may offer a novel and complementary perspective within the AOP computational toolkit.

Another novel element along with ToxAssay is our core gene identification method, which integrates pathway enrichment and PPI analysis. Unlike previous algorithms such as EnrichNet (Glaab *et al.* 2012) and NetPEA (Liu *et al.* 2017), our method focuses on identifying densely interconnected, biologically functional regions within the network rather than solely discovering new pathways. Instead of relying on a single community detection method like EBC (Girvan and Newman 2002), Walktrap (Clauset *et al.* 2004), or Fastgreedy (Pons and Latapy 2005), we employ a stacking algorithm that enhances core gene detection by minimizing noise and reducing false-positive interactions, which is crucial for pinpointing key genes in large and complex networks. As a result, we identified a core subnetwork comprising 26 genes with the highest interconnectivity and functional relevance. The predictive performance of this combined gene set was validated by achieving high AUC values and strong cross-correlations with external databases. Following a comprehensive review of our 26 CDEGs, informed by prior research, we propose a molecular mechanism underlying glutathione depletion (see Supplementary Discussion and Supplementary Fig. S13). These 26 CDEGs form an intricate network supporting glutathione’s essential roles in detoxification, antioxidant defense, and overall cellular health, offering insights into therapeutic strategies for drug-induced toxicity.

Efforts continue to enhance predictive models by identifying genomic markers that improve the precision of determining causes and mechanisms of deaths due to sudden incidents, overdoses, or drug administration, thus enriching the intersection of medical and forensic genetics (Wendt and Budowle 2020; Di Nunno *et al.* 2021). ToxAssay has the potential in post-mortem toxicological analyses, aiding in the determination of the cause and manner of drug-related deaths.

A significant limitation of our study is the selection of an adequate number of training compounds to study glutathione depletion due to the lack of available knowledge. It is recommended to use more compounds in the ToxAssay, if possible, to enhance efficiency in toxicity assessment. Another notable limitation is the lack of experimental validation for the results obtained using ToxAssay. Empirical validation is essential to confirm the efficacy of our methodology. Additionally, our research relies heavily on *in vivo* rat data, which poses a limitation as the results may not fully translate to human subjects. This underscores the need for additional research to evaluate the applicability of our findings to different species. Addressing these limitations is crucial to strengthen the robustness and relevance of our research.

## 4 Methods

### 4.1 Toxicogenomics data

Several toxicogenomics databases have emerged, with Open TG-GATEs (Igarashi *et al.* 2015), DrugMatrix (Ganter *et al.* 2006), and CTD (Davis *et al.* 2023) being the leading public resources that establish direct links between toxicity and gene expression data (Alexander-Dann *et al.* 2018).

In Open TG-GATEs, liver and kidney samples were collected *in vivo*, where gene expressions of triplicate samples were recorded at four distinct time points (3, 6, 9, and 24 hours) within each of the three dose levels (low, middle and high) after administering a single dose of the studied compound. Repeated doses were also available wherein gene expressions were measured at 4, 8, 15, and 29 days under the same dose levels after the initial administration. In addition, *in vitro* gene expressions in duplicate rats and human primary hepatocytes are available at three time points (2, 6, and 24 hours) and three dose levels (low, middle, and high).

The DrugMatrix dataset encompass various tissues, including liver, kidney, heart, and bone marrow. *In vivo* gene expressions of triplicate rat samples under repeat doses were recorded at 0.25, 1, 3, and 5 days (some replaced with 7/14/30/90 days) at two dose levels (low and high*). In vitro* gene expressions of duplicate rat primary hepatocytes were recorded at 16 and 24 hours after treatment with a high dose.

Additionally, ToxAssay utilizes the CTD to identify AOPs. The CTD is a curated collection of various links, including chemical-gene interactions, and chemical-disease and gene-disease associations. As of its most recent 2023 update, CTD encompasses 17 100 chemicals, 54 300 genes, 6100 phenotypes, 7270 diseases, and 202 000 exposure statements.

### 4.2 Hierarchical linear model (HLM)

Typical perturbation data of compounds in toxicogenomics exhibit a hierarchical, time-and-dose-dependent data structure (compound → dose → time point), revealing complex relationships among treatment, dosage, and time point (Supplementary Fig. S1). In a multilevel framework, gene expression values $y_{\mathrm{jklm}}$ of the *m*-th sample $\left(m=1,\;2,\cdots ,n_{\mathrm{jkl}}\right)$ at the *l*-th time point $\left(l=1,\;2,\;3,\ldots t_{jk}\;\right)$ following the administration of the *k*-th dose $\left(k=1,\;2,\;3,\cdots d_{j}\right)$ of the *j*-th compound (j=1, 2, 3…,b) are modeled as follows:


(1) yjklm=λjkl+εjklm,

the time point specific intercept ($\lambda_{\mathrm{jkl}}$) is given by:


(2) λjkl=γjk+ζjkl,

the dose specific intercept ($\gamma_{jk}$) is given by:


(3) γjk=βj+ξjk,

where $\beta_{j}$ is the intercept at the compound level.

Let $C_{1},C_{2},\ldots ,C_{a}$ be a set of compound groups (typically a=2, representing targeted toxicity-positive and toxicity-negative) to be compared for assessing targeted toxicity, where each $C_{i}$ contains $b_{i}$ compounds with similar toxicity characteristics. Then, the compound specific intercept ($\beta_{j}$) is given by:


(4) βj(i)=μ+τi+ηj, 

where μ is the general intercept and $\tau_{i}$ is the mean effect of the *i*-th compound group. It is assumed that the residuals $\varepsilon_{\mathrm{jklm}}$ are normally distributed, with a mean of zero and a variance of $\sigma_{\varepsilon }^{2}$, ie, $\varepsilon_{\mathrm{jklm}}∼N(0,\;\sigma_{\varepsilon }^{2})$. Just as the residuals $\varepsilon_{\mathrm{jklm}}$, the intercept residuals are $\zeta_{\mathrm{jkl}}∼N(0,\;\sigma_{\lambda }^{2})$, $\xi_{jk}∼N(0,\;\sigma_{\gamma }^{2})$ and $\eta_{j}∼N(0,\;\sigma_{\beta }^{2})$. It is assumed that the residuals across different levels are uncorrelated.

In multilevel modelling framework, ICC is a crucial statistical metric, quantifying the level of similarity within clustered data. The overall ICC is determined by $\rho =\sigma_{o}^{2}/(\sigma_{o}^{2}+\sigma_{\varepsilon }^{2})$, where ${\sigma_{o}^{2}=\sigma }_{\lambda }^{2}+\sigma_{\gamma }^{2}+\sigma_{\beta }^{2}$ is the total of intercept variances. When clustering is absent (ρ=0), all observations within compound-dose-time combinations are independent, that is, there is no variation between clusters and thus no dependency. Conversely, in the extreme case of ρ=1, observations within clusters are identical while those across clusters differ completely, representing total dependency.

### *4.3 F*-statistics of HLM for identifying DEGs

In a fixed-effect HLM, the hypotheses test the effect of all compound groups on gene expression values. The null hypothesis for the *F*-statistic assumes that the effects of all compound groups are absent ($H_{0}:\tau_{1}=\tau_{2}=\cdots =\tau_{a}=0$) adjusted for the extent of dependency in the data. The alternative hypothesis is that at least one $\tau_{i}\neq 0$. For each gene, the *F*-statistic can be derived as follows:


(5) F=∑ini(y¯i-y¯)2/(a−1)∑jklm(i)(yjklm(i)-y¯jkl(i))2/(n-p),

where $p=\sum_{i=1}^{a}\sum_{j=1}^{b_{i}}\sum_{k=1}^{d_{j(i)}}t_{jk(i)}$ is the total number of possible clusters in the data and $n_{i}=\sum_{j=1}^{b_{i}}\sum_{k=1}^{d_{j}}\sum_{l=1}^{t_{jk}}n_{\mathrm{jkl}}$ is total number of samples in the *i*-th group and total sample size using the summation rule is $n=\sum_{i=1}^{a}n_{i}=\sum_{i=1}^{a}\sum_{j=1}^{b_{i}}\sum_{k=1}^{d_{j(i)}}\sum_{l=1}^{t_{jk(i)}}n_{\mathrm{jkl}(i)}$ (for derivation see Supplementary Method B). Under the null, F has the central *F*-distribution with (a−1) and (n-p) degrees of freedom.

Let $\pi =\sum_{i}n_{i}{\left({\bar{y}}_{i}-\bar{y}\right)}^{2}$ and $\psi =\sum_{\mathrm{jklm}(i)}{\left(y_{\mathrm{jklm}(i)}-{\bar{y}}_{\mathrm{jkl}(i)}\right)}^{2}$, Equation (5) can be expressed as F=ηπψ, where η=(n-p)/(a−1). π and ψ can be calculated using the quadratic form as $\pi =y^{T}Ay$ and $\psi =y^{T}By$ (for derivation see Supplementary Method C), where $A={⊕}_{i=1}^{a}(\frac{1}{n_{i}}J_{n_{i}})-\frac{1}{n}J_{n}$ and $B=I_{n}-{⊕}_{i=1}^{a}{⊕}_{j=1}^{b_{i}}{⊕}_{k=1}^{d_{j(i)}}{⊕}_{l=1}^{t_{jk(i)}}\left(\frac{1}{n_{\mathrm{jkl}(i)}}J_{n_{\mathrm{jkl}(i)}}\right)$. The quadratic matrices A and B are not necessarily symmetric. $I_{n}$ is an identity matrix and $J_{n}$ is a square matrix of one of order n.

Let Y be the gene expression data matrix of dimension n×h; n is total number of samples and h is total number of genes to be tested. To derive the vector of all *F*-statistics $F_{g}(g=1,2,\;\cdots ,h)$ for all genes, instead of calculating *F*-values based on single gene expression values ($y_{\mathrm{jklm}}$) in loops, we show that matrix computation of Y could substantially reduce the computational burden. This is achieved as $F_{g}=\eta \Pi ⊙\Psi^{-1}$, where $\Pi ={\left(\pi_{1},\;\pi_{2},\;\ldots ,\;\pi_{h}\right)}^{T}$ and $\Psi ={\left(\psi_{1},\;\psi_{2},\;\ldots ,\;\psi_{h}\right)}^{T}$ be the vector of quadratic term for h genes. Π and Ψ can be calculated using Y as $\mathrm{diag}(Y^{T}AY)$ and $\mathrm{diag}(Y^{T}BY)$, respectively.

Let ${C=\{C}_{1},C_{2},\ldots ,C_{a}\}$ be the set of all compounds, where each $C_{i}$ contains $b_{i}$ compounds, then C can be written as $C=\;\{C_{i}=\{c_{ij}\;|\;j=1,\;2,\ldots ,b_{i}\},\;i=1,\;2,\;\cdots ,\;a\}$. Let the initial set of $h_{0}$ significant DEGs identified by the proposed $F_{g}$-test $\left(g=1,2,\cdots ,h_{0}\right)$, be denoted as $DE_{0}$, which may also include some genes influenced by chemical-specific outlying expressions. To remove these outlying genes from $DE_{0}$ set, we considered a leave ($b_{i}-1$)-out cross-validation framework for i=1,2,…,a, as discussed below. We generate $r=\sum_{i=1}^{a}b_{i}$ DEGs sets, denoted as $DE_{1},DE_{2},\ldots ,DE_{r}$, where each $DE_{j}(j=1,2,\ldots ,r)$ is computed using the proposed test statistic with the reduced set: $C^{-(b_{i}-1)}=\{c_{ij}|(b_{i}-1)\;\mathrm{compound}\;of\;C_{i}\notin C,\;j=1,2,\ldots ,r\}$, i=1,2,⋯,a. Finally, the outlier-free reduced DEGs set is obtained as: $\mathrm{DEGs}=\overset{r}{\underset{j=1}{\cap }}{DE}_{j}$.

### 4.4 Adverse outcome pathway (AOP) analysis

We developed an association rule mining algorithm to uncover significant correlations between gene targets in targeted toxicity and disease outcomes, with compounds acting as the linking mediators. Each rule within this framework comprises a trigger (gene) and an outcome (disease), suggesting genes playing a role in adverse outcomes. Here, each compound, along with its associated gene and disease interactions, is treated as a transaction.

Let $I=(I_{1},I_{2},\ldots ,I_{w})$ be set of items which includes both genes (G) and diseases (D), such that $I\in {(G}_{1},G_{2},\ldots G_{s},D_{1},D_{2},\ldots ,D_{q})$ and the set of the transactions for each compound is $T=(T_{1},T_{2},\ldots ,T_{r})$, where each $T_{i}\subseteq I$. We generate a transaction matrix $M=\{z_{ij},\;i=1,2,\ldots ,r;j=1,2,\ldots ,s+q\}$, where $z_{ij}=0$ or 1 is determined by the following:


$$z_{ij}=\{\begin{array}{cc} 1 & I_{j}\in T_{i}\\ 0 & I_{j}\notin T_{i} \end{array}.$$

After generating the transaction matrix using CTD database, similar to the previous study (Oki and Edwards 2016), we apply a quality control step to improve efficiency and accuracy by excluding genes and diseases with activity scores ≤ 1, ie, remove columns where $\sum_{i}z_{ij}\leq 1$.

In ToxAssay, we focused exclusively on pairwise associations between genes and diseases, considering only rules that involve one gene and one disease. The rule of the form $G_{s}\Rightarrow D_{q}$ satisfying $G_{s},D_{q}\subset I$ and $G_{s}\cap D_{q}=\emptyset$. To assess the strength of these associations, we used metrics like lift, support, confidence, and odds ratio (OR) (detailed in Supplementary Method D). We set a minimum support threshold of 0.3 transactions for rule generation. The choice of a 0.3 minimum support threshold for rule generation was selected to ensure that an association rule is identified only if the specific combination of gene expression changes and associated disease outcomes consistently occurs across a meaningful proportion (at least 30%) of the compounds analyzed. This threshold prioritizes robust and statistically significant biological relationships while minimizing the likelihood of identifying random or biologically irrelevant patterns. Such a criterion aligns with common data-mining practices in biological research that emphasize interpretability and reproducibility of results (Chen *et al.* 2015; Giulia *et al.* 2021; Qian and Sun 2023). Additionally, we selected association rules with a value of lift>1, indicating that the item is over-represented in the sample.

Furthermore, to assess disease-disease similarity, we employed the overlap score between diseases D1 and D2 (Goh *et al.* 2007):


$$\mathrm{sim}(D_{1},\;D_{2})=\frac{\left|G(D_{1})\cap G(D_{2})\right|}{\sqrt{\left|G(D_{1})|\times |G(D_{2})\right|}},$$

where G(·) represents the set of genes associated with each disease, and | · | denotes the cardinality of the set. Similarly, gene-gene similarity between genes G1 and G2 was calculated using:


$$\mathrm{sim}(G_{1},\;G_{2})=\frac{\left|D(G_{1})\cap D(G_{2})\right|}{\sqrt{\left|D(G_{1})|\times |D(G_{2})\right|}},$$

where D(·) represents the set of diseases associated with each gene. The pairwise similarity is used to compute a distance matrix as 1-sim. Genes or diseases are then hierarchically clustered, and clusters are obtained by cutting the tree at the desired threshold.

### 4.5 Functional enrichment and PPI network analysis

To reveal and compare the potential functional roles of the ToxAssay findings, we investigate the enrichment of identified gene signature in four pathway databases, including KEGG (Kanehisa *et al.* 2017), GO (Harris *et al.* 2004), Reactome (Croft *et al.* 2011) and Wikipathway (Slenter *et al.* 2018) using a false discovery rate (FDR) cutoff of 0.05. Additionally, the resulting GO terms were clustered into groups based on their semantic similarity, using Wang’s method to reduce redundancy in the GO lists (Wang *et al.* 2007).

To explore gene interactions, a PPI network was constructed using the STRING database with the latest available version 12.0, which contains more than 14 000 organisms and over 67 million proteins (Szklarczyk *et al.* 2021). The gene set was mapped to STRING to generate experimentally validated interactions among genes/proteins, with a score threshold of 200. Important modules and hub genes in the PPI network can be achieved by adjusting the centrality threshold, including eigenvector and degree. Three widely-used community detection algorithms for networks, namely edge betweenness centrality (EBC) (Girvan and Newman 2002), Walktrap (Clauset *et al.* 2004), and Fastgreedy (Pons and Latapy 2005), are applied to identify co-regulated subnetworks within the full network. The subnetworks containing less than 5% of the total proteins in the PPI network were combined into a unified miscellaneous subnetwork (SN0).

### 4.6 Identification of core DEGs (CDEGs)

We isolated key subnetworks from the PPI network to identify the most interconnected and biologically relevant genes from the DEG set, focusing on those with heightened biological and functional significance. Let $({SN}_{u1},{SN}_{u2},\ldots ,{SN}_{uv})\subseteq \mathrm{DEGs}$ be the set of genes (coding proteins) in the *v*-th subnetworks of the PPI network, obtained using the *u*-th community detection algorithm. The core genes are identified using the following formula:


$$\mathrm{CDEGs}=\underset{uv}{\cap }\left\{{SN}_{uv}|\frac{\left|E({SN}_{uv})\cap E(\mathrm{DEGs})\right|}{\left|E(\mathrm{DEGs})\right|}\geq \;\omega \right\},$$

where E(·) represents pathway enrichment in the database, and | · | denotes the cardinality of the set. In this study, we use the GO biological process, but users can select from various databases, including GO, KEGG, Reactome, or WikiPathways. The threshold ω (0≤ω≤1) represents the minimum percentage of the enrichment score that the subnetworks must capture.

### 4.7 Prediction model using gene signature

We evaluated the predictive capability of identified gene signature using both supervised and unsupervised learning algorithms, applied to both individual and average gene expression data. Often, researchers prefer to examine and compare mean gene expression values across different levels instead of focusing on individual data points. The average gene expression data for time point, dose, and compound levels are calculated efficiently as ${\bar{Y}}_{t}=Y^{T}\Omega_{t}$, ${\bar{Y}}_{d}=Y^{T}\Omega_{d}$, and ${\bar{Y}}_{c}=Y^{T}\Omega_{c}$, respectively, where $\Omega_{t}={⊕}_{j=1}^{b}{⊕}_{k=1}^{d_{j}}{⊕}_{l=1}^{t_{jk}}\left(\frac{1}{n_{\mathrm{jkl}}}1_{n_{\mathrm{jkl}}}\right)$, $\Omega_{d}={⊕}_{j=1}^{b}{⊕}_{k=1}^{d_{j}}\left(\frac{1}{n_{jk}}1_{n_{jk}}\right)$, and $\Omega_{c}={⊕}_{j=1}^{b}\left(\frac{1}{n_{j}}1_{n_{j}}\right)$ (for derivation see Supplementary Method E). Here, $n_{jk}=\sum_{l=1}^{t_{jk}}n_{\mathrm{jkl}}$, and $n_{j}=\sum_{k=1}^{d_{j}}\sum_{l=1}^{t_{jk}}n_{\mathrm{jkl}}$.

For the supervised classification analysis, logistic regression was employed to differentiate samples treated with glutathione-depleting (positive) versus non-glutathione-depleting (negative) compounds. The log_2_(FC) gene expression values served as independent variables. The dataset was balanced, containing seven compounds in each group, with each compound assessed across three dosages, four time points, and three replicates. This resulted in a total of 252 samples per group, and 504 samples in total. To avoid overfitting, logistic regression performance was evaluated through 100 repetitions of 10-fold cross-validation, with the overall predictive accuracy summarized as the mean AUC derived from these 1000 validation runs (100 repeats × 10 folds).

For the unsupervised hierarchical clustering analysis, Ward’s agglomerative method (“ward.D2” linkage) implemented in the R function *hclust* was applied (Murtagh and Legendre 2014). The analysis was conducted using data exclusively from the high-dose level, performed across all measured time points, and specifically for the maximum time points, where pathological alterations are most evident. Mean gene expression values across the three replicates per treatment condition were calculated and used. The optimal cluster number was determined primarily by dendrogram visual inspection, supplemented by silhouette scores for additional validation.

### 4.8 External validation of gene signature

To validate the ToxAssay gene signature, we performed a cross-correlation analysis comparing gene expression data from the Open TG-GATEs dataset with an external dataset from DrugMatrix. Specifically, we focused on Methimazole, a known hepatotoxicant associated with glutathione depletion. Methimazole was selected as it appears in both datasets, allowing for a direct comparison of gene expression perturbations in both experimental systems.

The analysis aimed to assess the consistency and robustness of the ToxAssay gene signature across independent toxicogenomic platforms. By comparing gene expression profiles in both datasets, we evaluated the generalizability of the ToxAssay signature in predicting glutathione depletion-related hepatotoxicity. The strong correlation in gene expression perturbations across datasets confirms the reliability and validity of the ToxAssay signature as a tool for identifying biomarkers and predicting drug-induced toxicity.

### 4.9 ToxAssay implementation and functionalities

ToxAssay was implemented as a user-friendly, publicly accessible, operating system independent R package, available at https://github.com/Fun-Gene/toxassay. Detailed information about its functionalities can be found in Supplementary Method A and package demonstration.

### 4.10 Simulations

We conducted extensive simulations to investigate the performance of ToxAssay in detecting gene signature under a variety of scenarios. The synthetic dataset was simulated in such a way that it imitates the structure of a toxicogenomics dataset. For each simulation scenario, we generated a gene expression dataset consisting of h genes from two independent compound groups: one with samples exposed to toxicity-positive compounds and the other to toxicity-negative compounds, to study targeted toxicity.

The compound exposure gene expression values of each data point were simulated to resemble *in vivo* single-dose data from Open TG-GATEs experiments. Therefore, the core of our simulations is to simulate the gene expression matrix Y of n samples and h genes comprising $h_{1}$ DEGs and $h_{2}$ non-DEGs, where $h=h_{1}+h_{2}$. The expression values for each gene were generated from a Gaussian distribution $y_{\mathrm{sim}}∼N(\lambda_{l(jk)},\;\sigma_{y}^{2})$, where the intercept of the time level was simulated from a Gaussian distribution $\lambda_{l(jk)}∼N(\gamma_{k(j)},\;\sigma_{\lambda }^{2})$, and the intercept of the dose level was simulated from $\gamma_{k(j)}∼N(\beta_{j},\;\sigma_{\gamma }^{2})$, and the intercept of the compound level was simulated from $\beta_{j}∼N(\mu +\tau_{i},\;\sigma_{\beta }^{2})$. The overall intercept (μ) across all compounds was simulated from the uniform distribution μ∼U(-θ,θ) and τ is defined using effect size (δ).

We used a standardized model where observations are normalized to a mean of 0 and standard deviation of 1, allowing the total of intercept variances ($\sigma_{o}^{2}$) approximate the overall ICC (ρ). The variance of intercepts $\sigma_{\beta }^{2}$, $\sigma_{\gamma }^{2}$ and $\sigma_{\lambda }^{2}$ were simulated from a uniform distribution, U(0, ρ) with the condition $\sigma_{o}^{2}=\rho$. As we used a standardized model, the residual variance $\sigma_{\varepsilon }^{2}$ was generated from Gaussian distribution $\sigma_{\varepsilon }^{2}∼N(0,\;\sigma_{\varepsilon }^{2})$ such that $\sigma_{\varepsilon }^{2}+\rho +r^{2}=1$, where the explained variance $r^{2}$ can be approximated by the effect size (δ) as: $r^{2}={\left(\frac{\delta }{\sqrt{\delta^{2}+4}}\right)}^{2}$(Aarts *et al.* 2014). Varying effect sizes were applied to a proportion (κ) of the h genes to establish true effects (τ) for $h_{1}\in (1,\;2,\;\cdots ,\left\lfloor \kappa h\right\rfloor )$ DEGs. For $h_{2}\in (\left\lceil \kappa h\right\rceil ,\;\left\lceil \kappa h\right\rceil +1,\;\cdots ,h)$ non-DEGs, we set δ=0 and assume that $\sigma_{o}^{2}\approx 0$ (genes are equally expressed across all levels).

### 4.11 Application of ToxAssay in glutathione depletion analysis

Glutathione, a tripeptide composed of glutamate, cysteine, and glycine, plays a central role in defending against the toxicity of drugs and oxidants (Dickinson and Forman 2002; Forman *et al.* 2009). Upon exposure to toxic compounds, glutathione is depleted through conjugation with these substances, impairing antioxidant defenses and increasing the risk of drug-induced hepatotoxicity and other adverse effects. Understanding this mechanism is essential for developing strategies to enhance antioxidant protection and reduce drug toxicity. Consequently, glutathione depletion remains a key endpoint in drug-induced liver injury and is recognized as an early indicator of hepatotoxicity during drug development.

To investigate biomarker genes and illuminate the functional and molecular mechanisms related to glutathione depletion, we selected two sets of compounds: one comprising compounds known to conjugate with or deplete glutathione (positive set), and the other consisting of compounds not influenced by glutathione levels (negative set). The positive compounds were acetaminophen, bromobenzene, coumarin, methapyrilene, nitrofurazone, phorone, and diethyl maleate; the negative set included erythromycin- ethylsuccinate, gentamicin, glibenclamide, hexachlorobenzene, isoniazid, penicillamine, and perhexiline. All compounds have been previously employed in glutathione depletion studies (Kiyosawa *et al.* 2004; 2007; Gao *et al.* 2010; Nyström-Persson *et al.* 2013; Hasan *et al.* 2018; Rana *et al.* 2018). Following a similar design approach to previous research, we utilized the “rat/*in vivo*/liver/single” dataset of compounds from the Open TG-GATEs database for glutathione depletion analysis. The dataset consists of seven compounds × three doses × four time points × three replicates, resulting in 252 samples in both the positive and negative groups, for a total of 504 samples. Details regarding the training compounds can be found in Supplementary Table S7.

## Supplementary Material

btaf561_Supplementary_Data

## Data Availability

In this study, we used data from publicly available sources. Data sources included the Open TG-GATEs: https://dbarchive.biosciencedbc.jp/en/open-tggates/data-2.html, DrugMatrix: https://ntp.niehs.nih.gov/data/drugmatrix, CTD: https://ctdbase.org/about/, STRING database: https://string-db.org.
