# Supplementary material for: ToxAssay: a hierarchical model-driven tool for advanced toxicogenomics biomarker discovery
Source: Bioinformatics. 2025 Oct 11;41(10):btaf561. doi: 10.1093/bioinformatics/btaf561 (PMC12571508; doi:10.1093/bioinformatics/btaf561)
Supplement: btaf561_Supplementary_Data [file btaf561_supplementary_data.zip › ToxAssay_SI.docx]

***Supplementary information***

**ToxAssay: A hierarchical model-driven tool for advanced toxicogenomics biomarker discovery**

Md. Masud Rana^1,2^, Md. Nurul Haque Mollah^2^, Mohammed H. Albujja^4^, Sibte Syed Hadi^4^, Fan Liu^4*^

1. National Genomics Data Center, Beijing Institute of Genomics, Chinese Academy of Sciences and China National Center for Bioinformation, Beijing 100101, China
2. University of Chinese Academy of Sciences, Beijing 100049, China
3. Bioinformatics Laboratory, Department of Statistics, University of Rajshahi, Rajshahi 6205, Bangladesh.
4. Department of Forensic Sciences, College of Criminal Justice, Naif Arab University for Security Sciences, Riyadh, Kingdom Saudi Arabia.

* Correspondence: FL [fliu@nauss.edu.sa](mailto:fliu@nauss.edu.sa)

# **Supplementary Note**

**ToxAssay flowchart**

The algorithm flowchart of ToxAssay is illustrated in Figure 1b. Consider compound groups $\mathbf{C}=(C_{1}, C_{2},\ldots,C_{a})$, selected for comparison to study targeted toxicity, where each $C_{i}$ contains $b_{i}$ compounds that exhibit similar toxicity properties. ToxAssay takes $\mathbf{C}$ as input, along with the gene expression matrix $\mathbf{Y}$, metadata $\mathbf{X}$ and compound-gene ($\mathbf{V}_{\mathbf{1}}$) and compound-disease ($\mathbf{V}_{\mathbf{2}}$) association data from the CTD.

The variance components for HLM, $\boldsymbol{\Pi}$ and $\boldsymbol{\Psi}$ in $\boldsymbol{F}_{g}$ are calculated with the functions get_pi(**C**, **A**, **Y**, **X**) and get_psi(**C**, **B**, **Y**, **X**) respectively. The quadratic matrices **A** and **B** for $\boldsymbol{\Pi}$ and $\boldsymbol{\Psi}$ respectively, are calculated using the functions get_matrix(**C**, **X**)**.** The core functions for identifying DEGs is tgx_genes(**C**, **Y**, **X**) which generate a table $\mathbf{Z}$ containing information about gene signature.

For AOP analysis, the function get_transaction(**V**_1_, **V**_2_, **Z**) generates the transaction matrix $\mathbf{M}$, and the core function get_aops(**M**) applies the proposed association rule mining algorithm to identify significant disease outcomes linked to gene signatures for targeted toxicity.

The data frame $\mathbf{Z}$ is subsequently utilized in other core functions of ToxAssay’s automated systems biology analysis, including get_enrichment(**Z**), get_ppinet(**Z**), and core_degs(**Z**). The functions get_enrichment(**Z**) and get_ppinet(**Z**) perform automated pathway enrichment and PPI network analysis of significant DEGs, respectively. The function core_degs(**Z**) identifies CDEGs.

Finally, the function get_subset(**C**, **Y**, **X**, D**,** T) is used to generate a data subset for a specified dose (D) and time point (T). This subset is then utilized to develop a machine learning classifier with the get_clasifier(**Z**, **Y**, **X**, D, T) function, which predicts the toxicity class of test samples.

# **Supplementary Discussion**

**Molecular mechanism underlying glutathione depletion**

Following a comprehensive review of our 26 CDEGs, informed by prior research, we propose a molecular mechanism underlying glutathione depletion (Supplementary Figure S13). These 26 CDEGs form an intricate network supporting glutathione’s essential roles in detoxification, antioxidant defense, and overall cellular health, as outlined below.

The synthesis of glutathione commences with the enzymatic activity of *Gclc* and *Gclm*, which catalyze the amalgamation of glutamate and cysteine, followed by the addition of glycine (Lu, 2009; Lu, 2013). The cystine/glutamate antiporter system (xCT), encompassing *Slc3a2*, *Slc7a11* and *Slc17a3*, is crucial in securing a steady cysteine supply for glutathione synthesis (Jyotsana, et al., 2022; Shin, et al., 2017). *Gsr* is indispensable in the glutathione recycling process, converting oxidized glutathione (GSSG) back to its reduced state (GSH) using NADPH produced by *G6pd* (McDonagh, et al., 2013). This recycling is critical for maintaining a consistent glutathione pool to mitigate oxidative stress.

In the detoxification cascade, glutathione S-transferases, including *Gstm1*, *Gstm4*, *Gsta3* and *Mgst2* play pivotal roles in attaching glutathione to xenobiotics, thereby aiding their expulsion (Ramsay and Dilda, 2014; Townsend and Tew, 2003). *Akr7a3* participates in phase II metabolism, enhancing detoxification capabilities (Ahmed, et al., 2011), whereas *Cyp1a2* is essential for xenobiotic metabolic processing (Faber, et al., 2005), further aiding in detoxification. *Ugdh* and *Ephx1* further process these conjugates (Price, et al., 2023; Václavíková, et al., 2015), with the multidrug resistance protein *Abcc4* being vital for their transportation and elimination (Klaassen and Lu, 2008).

In response to oxidative stress, *Nqo1* and *Hmox1* are instrumental in reducing damage by neutralizing harmful compounds (Forman and Zhang, 2021). *Srxn1*, *Txnrd1*, and *Txn1* are central to sustaining cellular redox equilibrium (Tebay, et al., 2015). *Atf4* orchestrates the expression of genes crucial for stress response (Fusakio, et al., 2016), while *Ftl1* and *Cp* are involved in iron homeostasis, influencing glutathione levels (Cronin, et al., 2019; Nishizawa, et al., 2020), and *Zfand2a* and *Hspb8* contribute to protein quality control and stress resistance (Cristofani, et al., 2021).

# **Supplementary Methods**

## **Supplementary Method A**

ToxAssay is implemented in a tidy R framework through an R package. ToxAssay utilizes a set of core functions for the comprehensive analysis of toxicogenomics data, making it a handy tool capable of effectively analyzing a wide range of toxicological endpoints. Consider a set of compound groups $\mathbf{C}=(C_{1}, C_{2},\cdots,C_{a})$ where typically $a=2$ and each $C_{i}$ exhibit similar toxicity properties, containing $\mathbf{b}=(b_{1},b_{2},\ldots,b_{a})$ compounds intended for comparison in the assessment of targeted toxicity. Let $\mathbf{Y}$ be the matrix of gene expression values with probes/genes in rows and samples in columns, and $\mathbf{X}$ be the data frame containing metadata with sample information. The metadata ($\mathbf{X}$**)** must include columns such as barcode/sample_id (matching the columns of $\mathbf{Y}$), compound_name, dose_level, time_level and replicate_id, to create design matrices and other necessary matrices. The input for most of the core function is either $(C_{1},C_{2},\ldots,C_{a}, \mathbf{Y}, \mathbf{X})$ or simply $(\mathbf{C}, \mathbf{Y}, \mathbf{X})$, where $\mathbf{C}$ is a list containing all the vector of compounds $C_{i}$.

**Download and preprocessing the compound perturbation data**

The raw data of compounds from Open TG-GATEs and DrugMatrix were downloaded from national bioscience database center (NBDC) (<https://dbarchive.biosciencedbc.jp/en/open-tggates/download.html>) and from national toxicology project (NTP) (<https://ntp.niehs.nih.gov/data/drugmatrix>) through GEO, respectively. To decrease the low-intensity noise and extract expression values from CEL-files, the **affy** (Gautier, et al., 2004) package for R in Bioconductor was utilized. The data were then normalized by the median values using the RMA algorithm and Affymetrix probes are mapped to Ensembl gene identifiers from custom cdf files, using R package **rat2302cdf** and **hgu133plus2cdf** for rat and human, respectively.

In toxicogenomics studies, to achieve greater clarity and consistency, it is common to use log_2_ fold change (FC) data of compounds instead of raw expression data (Hasan, et al., 2018; Mohsen, et al., 2021; Nyström-Persson, et al., 2013; Rana, et al., 2017). The log_2_ FC gene expression values of each sample are calculated by comparing the treatment condition (combination of compound, dose and time) with the corresponding control samples. To calculate the FC value for each treatment, the median value of the control samples is utilized to ensure more robust estimation, especially when dealing with small sample sizes where only triplicate samples are available in the control group. The log_2_ FC gene expression values $y$ for each sample were calculated from raw gene expression values $y^{'}$ and concurrent control samples $y_{0}$ as

$$\begin{aligned} y=log2\left( \frac{y^{'}}{med\left( y_{0} \right)} \right). \end{aligned}$$

Finally, the gene expression matrix ($\mathbf{Y}$) is constructed by compiling individual gene expression vectors, with probes arranged in rows and sample IDs or barcodes in columns. The corresponding metadata ($\mathbf{X}$) contains sample-specific information, including sample IDs or barcodes, compound names, dose levels, time points, and replicates. We developed a function, process_tgxdata(**path**, …), that requires only the file path where all raw CELL files are stored, along with a TSV, CSV, or text file containing the data dictionary with CELL file details. This function prepares the input data for the gene expression matrix ($\mathbf{Y}$) and the corresponding metadata ($\mathbf{X}$), making it ready for use with ToxAssay.

**Identification of significant genes**

The quadratic matrices $\mathbf{A}$ and $\mathbf{B}$ are generated using the function get_matrix(**C**, **X**) for $\pi$ and $\psi$, respectively. The functions get_pi(**Y**, **A**) and get_psi(**Y**, **B**) is used to estimate variance components $\boldsymbol{\Pi}$ and $\boldsymbol{\Psi}$ for genes. The function get_degs(**C**, **Y**, **X**) calculates the test statistic $\boldsymbol{F}_{h}$ and compares it with the theoretical distribution for all genes.

In the initial step, ToxAssay identify $g_{0}$ DEGs using get_degs(**C**, **Y**, **X**) Subsequently, it filtered out all genes showing equal expression from the dataset and identify a refined set of common genes $g_{s}$ significant across all $\mathbf{C}_{ij}$ using the function tgx_degs(**C**, **Y**, **X**). Finally, it provides a gene table $\mathbf{Z}$ with gene identifiers, groups FC differences, p-values, and information about differential expression. The process of identify final $g_{s}$ significant genes using the function tgx_degs(**C**, **Y**, **X**) is described below:

$$\boldsymbol{for} each group i\mathrm{in}\mathbf{C}\{$$

$$\boldsymbol{for} each compound j\mathrm{in}\mathbf{b} \{$$

$$\boldsymbol{if}\left( j \mathrm{in}C_{i} \right)\{C_{ij}=j\}$$

$$\boldsymbol{else} \{C_{ij}=C_{ij}\}\};$$

$$g_{j} = \boldsymbol{F}_{h}(\mathbf{C}_{ij})\};$$

$$g_{s}=\bigcap_{j=1}^{\left| \boldsymbol{b} \right|} g_{j};$$

$$\boldsymbol{return} g_{s};$$

**Adverse outcome pathways analysis (**get_aops()**)**

The training compounds $\mathbf{C}$ are utilized to curate interaction data pertaining to compound-gene ($\mathbf{V}_{1}$) and compound-disease ($\mathbf{V}_{2}$) associations from CTD. The function get_transaction(**V**_1_, **V**_2_,g_s_) is used to generate transaction matrix $\mathbf{M}$. The function get_aops(**M**) identifies disease phenotypes as AOPs in the CTD using Medical Subject Headings (MeSH) descriptors (<http://www.nlm.nih.gov/mesh/>) and generates a table (**O**) with metrics (support, confidence, lift, OR) to assess gene-disease association strength.

**Functional enrichment and protein-protein interaction (PPI) network analysis**

The R package **STRINGdb** for R interface to the STRING database (<https://string-db.org>) is utilized for functional enrichment and PPI analysis. The function get_enrichment(**Z**) is designed to perform pathway enrichment analysis by exploring pathways from KEGG, Reactome, WikiPathways, and the Gene Ontology (GO) categories, which include biological processes, molecular functions, and cellular components. It generates a pathway enrichment table (**P**) that lists significant pathways, p-values, involved genes, and other relevant information.

The function get_ppinet(**Z**) retrieves PPI network data for significant genes from the STRING database and identifies subnetworks using various community detection algorithms. It generates a network dataset (**N**) in the form of a list object, comprising edges and vertices. The vertices data frame contains information about the nodes, including the STRING protein ID, corresponding gene symbol, various centrality measures, and subnetwork cluster information. Additionally, the function get_hubdata(**N**) is specifically designed to identify hub genes or proteins within a dense network by utilizing a user-defined centrality algorithm and threshold value.

**Identification of core DEGs**

The function core_degs(**Z**, category = "go", enrich_cutoff = 0.25, path_n = NULL) is developed to identify core DEGs from a given gene signature. It leverages both get_enrichment**(Z)** and get_ppinet(**Z**) functions to identify crucial subnetworks that have higher functional relevance, subsequently reporting genes within these subnetworks as core DEGs. In addition to the primary input $\mathbf{Z}$, core_degs() considers several parameters: ‘*category*’ allows for the selection of a specific pathway database to identify functionally significant subnetworks; ‘*enrich_cutoff*’ sets the minimum enrichment score threshold; and ‘*path_n*’ determines the number of top significant pathways to be considered in identifying important subnetworks.

**Subsetting and averaging the gene expression data**

The function get_subset() allows users to determine the gene expression for specific combinations of dose and time, offering a comprehensive understanding of gene expression patterns, in relation to different treatment conditions. Let $D$ and $T$ represent the sets of dose and time points used for subsetting the gene expression data ($\mathbf{Y}$) and metadata ($\mathbf{X}$) data. The process of subsetting data using the function get_subset(**C**, **Y**, **X,** D**,** T) is described below:

$$\boldsymbol{for}each dose d in D\boldsymbol{\{}$$

$$\boldsymbol{for}each time t in T\boldsymbol{\{}$$

$\boldsymbol{if}\left( d in\boldsymbol{X}\left[ d \right]\boldsymbol{\&}t in\boldsymbol{X}\left[ t \right] \right)$***{***

$\boldsymbol{Y,X=Y,X(X}\left[ d\boldsymbol{,}t \right]\boldsymbol{)\}\}\}}$; *# Filter the data*

$$\boldsymbol{return Y,X;}$$

On the other hand, get_avgFC() enables the calculation of the average gene expression across various levels, providing valuable insights into how genes respond under different experimental conditions. get_avgFC(**C**, **Y**, **X)** calculates the average gene expressions using the above formulas and produces a tidy dataset of average FC gene expression values for all the factor levels.

**Toxicity prediction**

Numerous studies have selected to concentrate on data subsets that either focus on high doses or approach the maximum tolerable levels, often also narrowing their analysis to specific time points, for the purpose of classifier optimization. User can select subset of data using the function get_subset() to train a classifier. The function get_classifier(**C**, **Y**^’^, **X**^’^) provides a comprehensive selection of classifiers for model fitting, including logistic regression (the default), Naïve Bayes, k-Nearest Neighbors (k-NN), XGBoost, and Support Vector Machines (SVM). The transpose of the gene expression matrix ($\mathbf{Y}$) serves as the data/predictor matrix, while the class variable for the samples (or treatment conditions) is derived from the compound group ($\mathbf{C}$). For example, toxicity-positive compounds are labeled as “yes” (1) and toxicity-negative compounds as “no” (0).

**Visualization in ToxAssay**

ToxAssay offers a variety of functions for data visualization and analysis, in addition to its data processing capabilities. Detailed information about these functionalities can be found in the package’s demonstration (<https://github.com/Fun-Gene/ToxAssay>).

## **Supplementary Method B**

There are two ways to handle toxicogenomics data. The conventional method involves either averaging across all observations within each cluster or pooling observations into a higher-level cluster and applying standard statistical techniques like the *t*-test (or, ANOVA) to these means, treating them as independent observations. Alternatively, to avoid the data reduction inherent in such averaging, a multilevel model can be employed to better handle the hierarchical structure of the data. When $\rho>0$, it indicates similarity within clusters, suggesting that the effective number of observations is less than the total number of observations. This reduction in the effective number of observations can lead to either an overestimation or an underestimation of the standard error of the mean, thus impacting the statistical power and precision of inferential conclusions (Quené and Bergh, 2004).

If we consider only two groups of compounds, the model described in equation (4) of the main manuscript can be expressed as:

$$\beta_{j}=\mu+\tau Z_{j}+\eta_{j},$$

where $Z_{j}$ represents a dummy coded indicator variable at the group level, indicating the experimental condition of cluster $j$ (e.g., 1 for targeted toxicity-positive and 0 for toxicity-negative). Here, $\mu$ signifies the overall intercept, denoting the overall mean in the negative compounds group when the indicator variable $Z_{j}$ equals 0 for the non-toxic condition and $\tau$ denotes the overall deviation of the positive compounds group from the negative compounds group when the indicator variable $Z_{j}$ equals 1 for the toxic condition. Note that if $\sigma_{0}^{2}=0$, implying the absence of clustering, all subscripts except the compound group become redundant. In such a scenario, the model at the individual observation level simplifies to:

$$y_{im}=\mu+\tau Z_{m}+\varepsilon_{im}, i=1, 2; m=1, 2,\cdots, n,$$

essentially resembling the standard *t*-test expressed in regression terms. The *t*-statistic to test the hypothesis of the absence of a group effect, i.e., $H_{0}:\tau=0$, is expressed as:

$$\begin{aligned} t=\frac{\overline{y}_{1}-\overline{y}_{2}}{s\sqrt{\frac{1}{n_{1}}+\frac{1}{n_{2}}}},\#\left( 2 \right) \end{aligned}$$

where $\overline{y}_{1}$ and $\overline{y}_{2}$ are the sample means of toxicity-positive and toxicity-negative compound groups with sample size $n_{1}$ and $n_{2}$, respectively, and $s$ is the estimated standard error of the mean difference.

Let the gene expression values be denoted as $y_{jklm}$, representing the *m*-th sample replicate $\left( m=1, 2, \cdots, n_{jkl} \right)$ at the *l*-th time point $(l=1, 2, 3,\cdots t_{jk} )$, which are nested within the *k*-th dose $(k=1, 2, 3, \cdots d_{j})$ of the *j*-th compound $\left( j=1, 2, 3\ldots,b \right)$ (Supplementary Figure S1). Considering the hierarchical structure of the data, let $y_{jklm(1)}$ and $y_{jklm(2)}$ represent samples from positive groups with $b_{1}$ compounds and negative group with $b_{2}$ compounds, respectively. The total sample sizes in the groups with targeted toxicity-positive and negative compounds are therefore $n_{1}=\sum_{j=1}^{b_{1}} \sum_{k=1}^{d_{j}} \sum_{l=1}^{t_{jk}} n_{jkl(1)}$ and $n_{2}=\sum_{j=1}^{b_{2}} \sum_{k=1}^{d_{j}} \sum_{l=1}^{t_{jk}} n_{jkl(2)}$, respectively. The test statistic in equation (2) can be simplified for HLM as:

$$\begin{aligned} t^{'}=\frac{\sqrt{\tilde{n}}\left( \overline{y}_{1}-\overline{y}_{2} \right)}{s^{'}},\#\left( 3 \right) \end{aligned}$$

where, $\tilde{n}$ is defined based on the total number of samples in targeted toxicity-positive and negative compound groups as $\tilde{n}={n_{1}n_{2}}/n$, where $n=n_{1}+n_{2}$ is the total sample size. When the treatments are assigned at the sample level and $\rho\geq0$, the standard error $s^{'}$ can be estimated as

$$\begin{aligned} s^{'}=\sqrt{\frac{\left( n_{1}-n_{1}/n_{0} \right)\hat{s}_{1}^{2}+\left( n_{2}-n_{2}/n_{0} \right)\hat{s}_{2}^{2}}{n_{1}+n_{2}-\left( n_{1}+n_{2} \right)/n_{0}}},\#\left( 4 \right) \end{aligned}$$

where $n_{jkl}=n_{0}$ for equal replicates and $\hat{s}_{i}^{2}=\frac{\sum_{jklm} \left( y_{jklm(i)}-\bar{y}_{jkl(i)} \right)^{2}}{n_{i}-n_{i}/n_{0}}$ is the estimated variance of *i*-th sample group (Konstantopoulos, 2006; Konstantopoulos, 2008). Here, $\bar{y}_{jkl}$ is the sample mean of *l*-th time at the *k*-th dose of the *j*-th compound.

We can generalize the test for multiple compounds groups using the relation $t^{2}=F$ from one-way ANOVA model. The square of the numerator of equation (3) is

$$\tilde{n}\left( \overline{y}_{1}-\overline{y}_{2} \right)^{2}$$

$$=\frac{n_{1}n_{2}n}{n^{2}}\left( \overline{y}_{1}-\overline{y}_{2} \right)^{2}$$

$$=\frac{n_{1}n_{2}\left( n_{1}+n_{2} \right)}{n^{2}}\left( \overline{y}_{1}-\overline{y}_{2} \right)^{2}$$

$$=\left[ \frac{n_{1}n_{2}^{2}}{n^{2}}+\frac{n_{2}n_{1}^{2}}{n^{2}} \right]\left( \overline{y}_{1}-\overline{y}_{2} \right)^{2}$$

$$=\frac{n_{1}n_{2}^{2}}{{nn}^{2}}\left( \overline{y}_{1}-\overline{y}_{2} \right)^{2}+\frac{n_{2}n_{1}^{2}}{n^{2}}\left( \overline{y}_{1}-\overline{y}_{2} \right)^{2}$$

$$=n_{1}\left[ \frac{n_{2}\left( \overline{y}_{1}-\overline{y}_{2} \right)}{n} \right]^{2}+n_{2}\left[ \frac{n_{1}\left( \overline{y}_{1}-\overline{y}_{2} \right)}{n} \right]^{2}$$

$$=n_{1}\left[ \frac{n_{2}\overline{y}_{1}-n_{2}\overline{y}_{2}}{n} \right]^{2}+n_{2}\left[ \frac{n_{1}\overline{y}_{1}-n_{1}\overline{y}_{2}}{n} \right]^{2}$$

$$=n_{1}\left[ \frac{\left( n-n_{1} \right)\overline{y}_{1}-n_{2}\overline{y}_{2}}{n} \right]^{2}+n_{2}\left[ \frac{n_{1}\overline{y}_{1}-\left( n-n_{2} \right)\overline{y}_{2}}{n} \right]^{2}$$

$$=n_{1}\left[ \frac{n\overline{y}_{1}}{n}-\frac{n_{1}\overline{y}_{1}+n_{2}\overline{y}_{2}}{n_{1}+n_{2}} \right]^{2}+n_{2}\left[ \frac{n\overline{y}_{2}}{n}-\frac{n_{1}\overline{y}_{1}+n_{2}\overline{y}_{2}}{n_{1}+n_{2}} \right]^{2}$$

$$=n_{1}\left( \overline{y}_{1}-\bar{y} \right)^{2}+n_{2}\left( \overline{y}_{2}-\bar{y} \right)^{2}$$

$$=\sum_{i} n_{i}\left( \overline{y}_{i}-\bar{y} \right)^{2}.$$

The square of the expression in equation (4) is

$${s^{'}}^{2}=\frac{\sum_{jklm} \left( y_{jklm(1)}-\bar{y}_{jkl(1)} \right)^{2}+\sum_{jklm} \left( y_{jklm(2)}-\bar{y}_{jkl(2)} \right)^{2}}{n_{1}+n_{2}-\left( n_{1}+n_{2} \right)/n_{0}}$$

$$=\frac{\sum_{jklm(i)} \left( y_{jklm(i)}-\overline{y}_{jkl(i)} \right)^{2}}{n-n/n_{0}}$$

$$=\frac{\sum_{jklm(i)} \left( y_{jklm(i)}-\overline{y}_{jkl(i)} \right)^{2}}{n-\sum_{i=1}^{2} \sum_{j=1}^{b_{i}} \sum_{k=1}^{d_{j(i)}} \sum_{l=1}^{t_{jk(i)}} n_{jkl(i)}/n_{jkl(i)}}$$

$$=\frac{\sum_{jklm(i)} \left( y_{jklm(i)}-\overline{y}_{jkl(i)} \right)^{2}}{n-\sum_{i=1}^{2} \sum_{j=1}^{b_{i}} \sum_{k=1}^{d_{j(i)}} t_{jk(i)}}$$

$$=\frac{\sum_{jklm(i)} \left( y_{jklm(i)}-\overline{y}_{jkl(i)} \right)^{2}}{n-p},$$

where $p=\sum_{i=1}^{2} \sum_{j=1}^{b_{i}} \sum_{k=1}^{d_{j(i)}} t_{jk(i)}$. Now, the square of t-statistic is

$$t^{2}=\frac{\sum_{i} n_{i}\left( \overline{y}_{i}-\overline{y} \right)^{2}/(2-1)}{\sum_{jklm(i)} \left( y_{jklm(i)}-\overline{y}_{jkl(i)} \right)^{2}/n-p}.$$

Let, $a$ be the total number of compound groups then the above equation can be expressed as

$$F=\frac{\sum_{i} n_{i}\left( \overline{y}_{i}-\overline{y} \right)^{2}/(a-1)}{\sum_{jklm(i)} \left( y_{jklm(i)}-\overline{y}_{jkl(i)} \right)^{2}/(n-p)}.$$

## **Supplementary Method C**

**Derivation of** $\pi\mathbf{=}\boldsymbol{y}^{T}\mathbf{A}\boldsymbol{y}$**:**

We have,

$$\pi=\sum_{i=1}^{a} n_{i}\left( \overline{y}_{i}-\overline{y} \right)^{2}$$

$$=\sum_{i=1}^{a} n_{i}\left( \overline{y}_{i}^{2}-2\overline{y}_{i}\overline{y}+\overline{y}^{2} \right)$$

$$=\sum_{i=1}^{a} n_{i}\overline{y}_{i}^{2}-2\overline{y}\sum_{i=1}^{a} n_{i}\overline{y}_{i}+\sum_{i=1}^{a} n_{i}\overline{y}^{2}$$

$$=\sum_{i=1}^{a} n_{i}\overline{y}_{i}^{2}-2\overline{y}n\overline{y}+n\overline{y}^{2}$$

$$=\sum_{i=1}^{a} n_{i}\frac{Y_{i}^{2}}{n_{i}^{2}}-n\frac{Y^{2}}{n^{2}}$$

$$=\sum_{i=1}^{a} \frac{Y_{i}^{2}}{n_{i}}-\frac{Y^{2}}{n}$$

$$=\left( \frac{Y_{1}^{2}}{n_{1}}+\cdots+\frac{Y_{a}^{2}}{n_{a}} \right)-\frac{Y^{2}}{n}$$

$$=\boldsymbol{y}^{T}\left[ \begin{matrix} \frac{1}{n_{1}}\mathbf{J}_{n_{1}} & \mathbf{0}_{n_{1}, n-n_{1}} \\ \mathbf{0}_{n-n_{1}, n_{1}} & \mathbf{0}_{n-n_{1}} \end{matrix} \right]\boldsymbol{y}+\cdots+\boldsymbol{y}^{T}\left[ \begin{matrix} \mathbf{0}_{n-n_{a}} & \mathbf{0}_{n-n_{a},n_{a}} \\ \mathbf{0}_{n_{a}, n-n_{a}} & \frac{1}{N_{a}}\mathbf{J}_{n_{a}} \end{matrix} \right]\boldsymbol{y}-\boldsymbol{y}^{T}\left[ \frac{1}{n}\mathbf{J}_{n} \right]\boldsymbol{y}$$

$$=\boldsymbol{y}^{T}\left[ \begin{matrix} \frac{1}{n_{1}}\mathbf{J}_{n_{1}} & \cdots& 0 \\ \vdots& \ddots& \vdots\\ 0 & \cdots& \frac{1}{n_{a}}\mathbf{J}_{n_{a}} \end{matrix} \right]\boldsymbol{y}-\boldsymbol{y}^{T}\left[ \frac{1}{n}\mathbf{J}_{n} \right]\boldsymbol{y}$$

$$=\boldsymbol{y}^{T}\left[ \bigoplus_{i=1}^{a} \left( \frac{1}{n_{i}}\mathbf{J}_{n_{i}} \right) \right]y-y^{T}\left[ \frac{1}{n}\mathbf{J}_{n} \right]\boldsymbol{y}$$

$$=\boldsymbol{y}^{T}\left[ \bigoplus_{i=1}^{a} \left( \frac{1}{n_{i}}\mathbf{J}_{n_{i}} \right)-\frac{1}{n}\mathbf{J}_{n} \right]\boldsymbol{y}{=\boldsymbol{y}}^{T}\mathbf{A}\boldsymbol{y},$$

where $\mathbf{A}=\oplus_{i=1}^{a}\left( \frac{1}{n_{i}}\mathbf{J}_{n_{i}} \right)-\frac{1}{n}\mathbf{J}_{n}$ and $\mathbf{J}_{n}$ is square matrix of one of order *n*.

**Derivation of** $\psi\mathbf{=}\boldsymbol{y}^{T}\mathbf{B}\boldsymbol{y}$:

$$\psi=\sum_{jklm(i)} \left( y_{jklm(i)}-\overline{y}_{jkl(i)} \right)^{2}$$

$$=\sum_{jklm(i)} y_{jklm(i)}^{2}-2y_{jklm(i)}\overline{y}_{jkl(i)}+{\overline{y}_{jkl(i)}}^{2}$$

$$=\sum_{jklm(i)} \left( y_{jklm(i)}^{2}-2y_{jklm(i)}\overline{y}_{jkl(i)}+{\overline{y}_{jkl(i)}}^{2} \right)$$

$$=\sum_{jklm(i)} y_{jklm(i)}^{2}-2\sum_{jklm(i)} y_{jklm(i)}\overline{y}_{jkl(i)}+\sum_{jklm(i)} \overline{y}_{jkl(i)}^{2}$$

$$=\sum_{jklm(i)} y_{ijklm}^{2}-2\sum_{jklm(i)} y_{jklm(i)}\frac{Y_{jkl(i)}}{n_{jkl(i)}}+\sum_{jklm(i)} \frac{Y_{jkl(i)}^{2}}{n_{jkl(i)}^{2}}$$

$$=\sum_{jklm(i)} y_{jklm(i)}^{2}-2\sum_{jkl(i)} \frac{Y_{jkl(i)}^{2}}{n_{jkl(i)}}+\sum_{jkl(i)} n_{jkl(i)}\frac{Y_{jkl(i)}^{2}}{n_{jkl(i)}^{2}}$$

$$=\sum_{i=1}^{a} \sum_{j=1}^{b_{i}} \sum_{k=1}^{d_{j(i)}} \sum_{l=1}^{t_{jk(i)}} \sum_{m=1}^{n_{jkl(i)}} y_{jklm(i)}^{2}-\sum_{i=1}^{a} \sum_{j=1}^{b_{i}} \sum_{k=1}^{d_{j(i)}} \sum_{l=1}^{t_{jk(i)}} \frac{Y_{jkl(i)}^{2}}{n_{jkl(i)}}.$$

The above expression can be written similar to derivation of $\pi$ as

$$\boldsymbol{y}^{T}\mathbf{I}_{N}\boldsymbol{y}-\boldsymbol{y}\left[ \bigoplus_{i=1}^{a} \bigoplus_{j=1}^{b_{i}} \bigoplus_{k=1}^{d_{j(i)}} \bigoplus_{l=1}^{t_{jk(i)}} \frac{1}{n_{jkl(i)}}\mathbf{J}_{n_{jkl(i)}} \right]\boldsymbol{y}$$

$$=\boldsymbol{y}\left[ \mathbf{I}_{n}-\bigoplus_{i=1}^{a} \bigoplus_{j=1}^{b_{i}} \bigoplus_{k=1}^{d_{j(i)}} \bigoplus_{l=1}^{t_{jk(i)}} \frac{1}{n_{jkl(i)}}\mathbf{J}_{n_{jkl(i)}} \right]\boldsymbol{y}=\boldsymbol{y}^{T}\mathbf{B}\boldsymbol{y},$$

where $\mathbf{B}=\mathbf{I}_{n}-\bigoplus_{i=1}^{a} \bigoplus_{j=1}^{b_{i}} \bigoplus_{k=1}^{d_{j(i)}} \bigoplus_{l=1}^{t_{jk(i)}} \frac{1}{n_{ijkl}}\mathbf{J}_{n_{ijkl}}$ and $\mathbf{I}_{n}$ is the identity of order *n*.

## **Supplementary Method D**

We employ various metrics, include support, confidence, lift, and the odds ratio (OR) to assess the association between specific genes and diseases in AOPs analysis.

**Support**: The support measures the frequency of simultaneous occurrences of a specific gene and disease within the dataset. A rule $G_{s}\Rightarrow D_{q}$ holds within the transaction set *T* with support *s*, where *s* represents the proportion of transactions in *T* that contain both $G_{s}$ and $D_{q}$, i.e., $G_{s}\cup D_{q}$. Mathematically, this is expressed as:

$$supp\left( G_{s}\Rightarrow D_{q} \right)=P\left( G_{s}\cup D_{q} \right).$$

Note that $P(G_{s}\cup D_{q})$ indicates the probability that a transaction contains the union of sets $G_{s}$ and $D_{q}$, not that a transaction contains either $G_{s}$ or $D_{q}$.

**Confidence**: Confidence assesses how frequently the disease is observed in instances where the gene is present, effectively capturing the conditional probability of observing the disease given the gene. For a rule $G_{s}\Rightarrow D_{q}$, the confidence *c* in the transaction set *T* is the proportion of transactions containing $G_{s}$ that also contain $D_{q}$, equivalent to the conditional probability of $D_{q}$ given $G_{s}$:

$$conf(G_{s}\Rightarrow D_{q})=P(D_{q}|G_{s})=\frac{supp\left( G_{s}\cup D_{q} \right)}{supp\left( G_{s} \right)}.$$

**Lift**: Lift offers a measure of the strength of an association by comparing the observed co-occurrence of a gene and a disease against what would be expected if they were statistically independent. The lift *l* for a rule $G_{s}\Rightarrow D_{q}$ is the ratio of the observed support to the expected support assuming independence:

$$lift(G_{s}\Rightarrow D_{q})=\frac{supp\left( G_{s}\cup D_{q} \right)}{supp(G_{s})supp(D_{q})}=\frac{conf\left( G_{s}\cup D_{q} \right)}{supp(D_{q})}.$$

**Odds Ratio (OR)**: The OR quantifies the likelihood of the disease occurring in the presence of the gene relative to its absence, providing insight into the strength of the association. The OR for a rule $G_{s}\Rightarrow D_{q}$ calculates the odds of $D_{q}$ occurring given $G_{s}$ versus its occurrence in the absence of $G_{s}$:

$$OR(G_{s}\Rightarrow D_{q})=\frac{odds\left( D_{q}|G_{s} \right)}{odds(D_{q}|\bar{G}_{s})}=\frac{supp\left( G_{s}\cup D_{q} \right)\times supp\left( \bar{G}_{s}\cup\bar{D}_{q} \right)}{supp\left( G_{s}\cup\bar{D}_{q} \right)\times supp\left( \bar{G}_{s}\cup D_{q} \right)}$$

## **Supplementary Method E**

**Derivation of** ${\bar{\mathbf{Y}}}_{\boldsymbol{c}}\mathbf{=}\mathbf{Y}^{\boldsymbol{T}}\boldsymbol{\Omega}_{\boldsymbol{c}}\mathbf{:}$

The average gene expression value at compound levels of a gene is calculated as

$$\bar{y}_{c}^{T}=\left[ \begin{matrix} \bar{y}_{1} & \cdots& \bar{y}_{b} \end{matrix} \right]$$

$$=\left[ \begin{matrix} \frac{Y_{1}}{n_{1}} & \cdots& \frac{Y_{b}}{n_{b}} \end{matrix} \right]$$

$$=\left[ \begin{matrix} \sum_{klm} \frac{y_{1klm}}{n_{1kl}} & \cdots& \sum_{klm} \frac{y_{bklm}}{n_{bkl}} \end{matrix} \right]$$

$$=\boldsymbol{y}^{T}\left[ \begin{matrix} \frac{1}{n_{1}}\mathbf{1}_{n_{1}} & \cdots& 0 \\ \vdots& \ddots& \vdots\\ 0 & \cdots& \frac{1}{n_{b}}\mathbf{1}_{n_{b}} \end{matrix} \right]$$

$$=\boldsymbol{y}^{T}\left[ \bigoplus_{j=1}^{b} \left( \frac{1}{n_{j}}1_{n_{j}} \right) \right]$$

$$=\boldsymbol{y}^{T}\Omega_{c}$$

Using vectorization, the average gene expression values at compound levels for a matrix of genes $\mathbf{Y}$ are efficiently calculated as ${\bar{\mathbf{Y}}}_{c}=\mathbf{Y}^{T}\Omega_{c}$.

**Derivation of** ${\bar{\mathbf{Y}}}_{\boldsymbol{d}}\boldsymbol{=}\mathbf{Y}^{\boldsymbol{T}}\boldsymbol{\Omega}_{\boldsymbol{d}}:$

The average gene expression value at dose levels of a gene is calculated as

$$\bar{y}_{d}^{T}=\left[ \begin{matrix} \bar{y}_{11} & \cdots& \bar{y}_{{bd}_{b}} \end{matrix} \right]$$

$$=\left[ \begin{matrix} \frac{Y_{11}}{n_{11}} & \cdots& \frac{Y_{{bd}_{b}}}{n_{{bd}_{b}}} \end{matrix} \right]$$

$$=\left[ \begin{matrix} \sum_{lm} \frac{y_{11lm}}{n_{11l}} & \cdots& \sum_{lm} \frac{y_{{bd}_{b}lm}}{n_{{bd}_{b}l}} \end{matrix} \right]$$

$$=\boldsymbol{y}^{T}\left[ \begin{matrix} \frac{1}{n_{11}}\mathbf{1}_{n_{11}} & \cdots& 0 \\ \vdots& \ddots& \vdots\\ 0 & \cdots& \frac{1}{n_{{bd}_{b}}}\mathbf{1}_{n_{{bd}_{b}}} \end{matrix} \right]$$

$$=\boldsymbol{y}^{T}\left[ \bigoplus_{j=1}^{b} \bigoplus_{k=1}^{d_{j}} \left( \frac{1}{n_{jk}}1_{n_{jk}} \right) \right]$$

$$=\boldsymbol{y}^{T}\Omega_{d}$$

Using vectorization, the average gene expression values at dose levels for a matrix of genes $\mathbf{Y}$ are efficiently calculated as ${\bar{\mathbf{Y}}}_{d}=\mathbf{Y}^{T}\Omega_{d}$.

**Derivation of** ${\bar{\mathbf{Y}}}_{\boldsymbol{t}}\boldsymbol{=}\mathbf{Y}^{\boldsymbol{T}}\boldsymbol{\Omega}_{\boldsymbol{t}}:$

The average gene expression value at time levels of a gene is calculated as

$$\bar{y}_{t}^{T}=\left[ \begin{matrix} \bar{y}_{111} & \cdots& \bar{y}_{bd_{b}t_{bd}} \end{matrix} \right]$$

$$=\left[ \begin{matrix} \frac{Y_{111}}{n_{111}} & \cdots& \frac{Y_{bd_{b}t_{bd}}}{n_{bd_{b}t_{bd}}} \end{matrix} \right]$$

$$=\left[ \begin{matrix} \sum_{m} \frac{y_{111m}}{n_{111}} & \cdots& \sum_{m} \frac{y_{bd_{b}t_{bd}m}}{n_{bd_{b}t_{bd}}} \end{matrix} \right]$$

$$=\boldsymbol{y}^{T}\left[ \begin{matrix} \frac{1}{n_{111}}\mathbf{1}_{n_{111}} & \cdots& 0 \\ \vdots& \ddots& \vdots\\ 0 & \cdots& \frac{1}{n_{bd_{b}t_{bd}}}\mathbf{1}_{bd_{b}t_{bd}} \end{matrix} \right]$$

$$=\boldsymbol{y}^{T}\left[ \bigoplus_{j=1}^{b} \bigoplus_{k=1}^{d_{j}} \bigoplus_{l=1}^{t_{jk}} \left( \frac{1}{n_{jkl}}1_{n_{jkl}} \right) \right]$$

$$=\boldsymbol{y}^{T}\Omega_{t}$$

Using vectorization, the average gene expression values at time levels for a matrix of genes $\mathbf{Y}$ are efficiently calculated as ${\bar{\mathbf{Y}}}_{t}=\mathbf{Y}^{T}\Omega_{t}$.

# **Supplementary References**

Ahmed, M.M.*, et al.* Aldo-keto reductase-7A protects liver cells and tissues from acetaminophen-induced oxidative stress and hepatotoxicity. *Hepatology* 2011;54(4):1322-1332.

Bates, D.*, et al.* Fitting Linear Mixed-Effects Models Using lme4. *Journal of Statistical Software* 2015;67(1):1 - 48.

Cristofani, R.*, et al.* The Role of HSPB8, a Component of the Chaperone-Assisted Selective Autophagy Machinery, in Cancer. *Cells* 2021;10(2).

Cronin, S.J.F.*, et al.* The Role of Iron Regulation in Immunometabolism and Immune-Related Disease. *Front Mol Biosci* 2019;6:116.

Faber, M.S., Jetter, A. and Fuhr, U. Assessment of CYP1A2 Activity in Clinical Practice: Why, How, and When? *Basic & Clinical Pharmacology & Toxicology* 2005;97(3):125-134.

Forman, H.J. and Zhang, H. Targeting oxidative stress in disease: promise and limitations of antioxidant therapy. *Nat Rev Drug Discov* 2021;20(9):689-709.

Fusakio, M.E.*, et al.* Transcription factor ATF4 directs basal and stress-induced gene expression in the unfolded protein response and cholesterol metabolism in the liver. *Mol Biol Cell* 2016;27(9):1536-1551.

Gautier, L.*, et al.* affy--analysis of Affymetrix GeneChip data at the probe level. *Bioinformatics* 2004;20(3):307-315.

Hasan, M.N.*, et al.* Robust Co-clustering to Discover Toxicogenomic Biomarkers and Their Regulatory Doses of Chemical Compounds Using Logistic Probabilistic Hidden Variable Model. *Front Genet* 2018;9:516.

Jyotsana, N., Ta, K.T. and DelGiorno, K.E. The Role of Cystine/Glutamate Antiporter SLC7A11/xCT in the Pathophysiology of Cancer. *Front Oncol* 2022;12:858462.

Klaassen, C.D. and Lu, H. Xenobiotic transporters: ascribing function from gene knockout and mutation studies. *Toxicol Sci* 2008;101(2):186-196.

Konstantopoulos, S. The Power of the Test in Three-Level Designs. In.: Institute of Labor Economics (IZA); 2006.

Konstantopoulos, S. The Power of the Test for Treatment Effects in Three-Level Cluster Randomized Designs. *Journal of Research on Educational Effectiveness* 2008;1(1):66-88.

Lu, S.C. Regulation of glutathione synthesis. *Mol Aspects Med* 2009;30(1-2):42-59.

Lu, S.C. Glutathione synthesis. *Biochim Biophys Acta* 2013;1830(5):3143-3153.

McDonagh, E.M.*, et al.* PharmGKB summary: methylene blue pathway. *Pharmacogenet Genomics* 2013;23(9):498-508.

Mohsen, A., Tripathi, L.P. and Mizuguchi, K. Deep Learning Prediction of Adverse Drug Reactions in Drug Discovery Using Open TG–GATEs and FAERS Databases. *Frontiers in Drug Discovery* 2021;1.

Nishizawa, H.*, et al.* Ferroptosis is controlled by the coordinated transcriptional regulation of glutathione and labile iron metabolism by the transcription factor BACH1. *J Biol Chem* 2020;295(1):69-82.

Nyström-Persson, J.*, et al.* Toxygates: interactive toxicity analysis on a hybrid microarray and linked data platform. *Bioinformatics* 2013;29(23):3080-3086.

Price, M.J.*, et al.* UDP-glucose dehydrogenase (UGDH) in clinical oncology and cancer biology. *Oncotarget* 2023;14:843-857.

Quené, H. and Bergh, H.v.d. On multi-level modeling of data from repeated measures designs: a tutorial. *Speech Commun.* 2004;43:103-121.

Ramsay, E.E. and Dilda, P.J. Glutathione S-conjugates as prodrugs to target drug-resistant tumors. *Front Pharmacol* 2014;5:181.

Rana, M.*, et al.* A novel computational approach for toxicogenomics biomarker discovery in drug development pipeline. *Journal of Bio-Science* 2017;25:57-66.

Shin, C.S.*, et al.* The glutamate/cystine xCT antiporter antagonizes glutamine metabolism and reduces nutrient flexibility. *Nat Commun* 2017;8:15074.

Tebay, L.E.*, et al.* Mechanisms of activation of the transcription factor Nrf2 by redox stressors, nutrient cues, and energy status and the pathways through which it attenuates degenerative disease. *Free Radic Biol Med* 2015;88(Pt B):108-146.

Townsend, D.M. and Tew, K.D. The role of glutathione-S-transferase in anti-cancer drug resistance. *Oncogene* 2003;22(47):7369-7375.

Václavíková, R., Hughes, D.J. and Souček, P. Microsomal epoxide hydrolase 1 (EPHX1): Gene, structure, function, and role in human disease. *Gene* 2015;571(1):1-8.

# **Supplementary Figures**


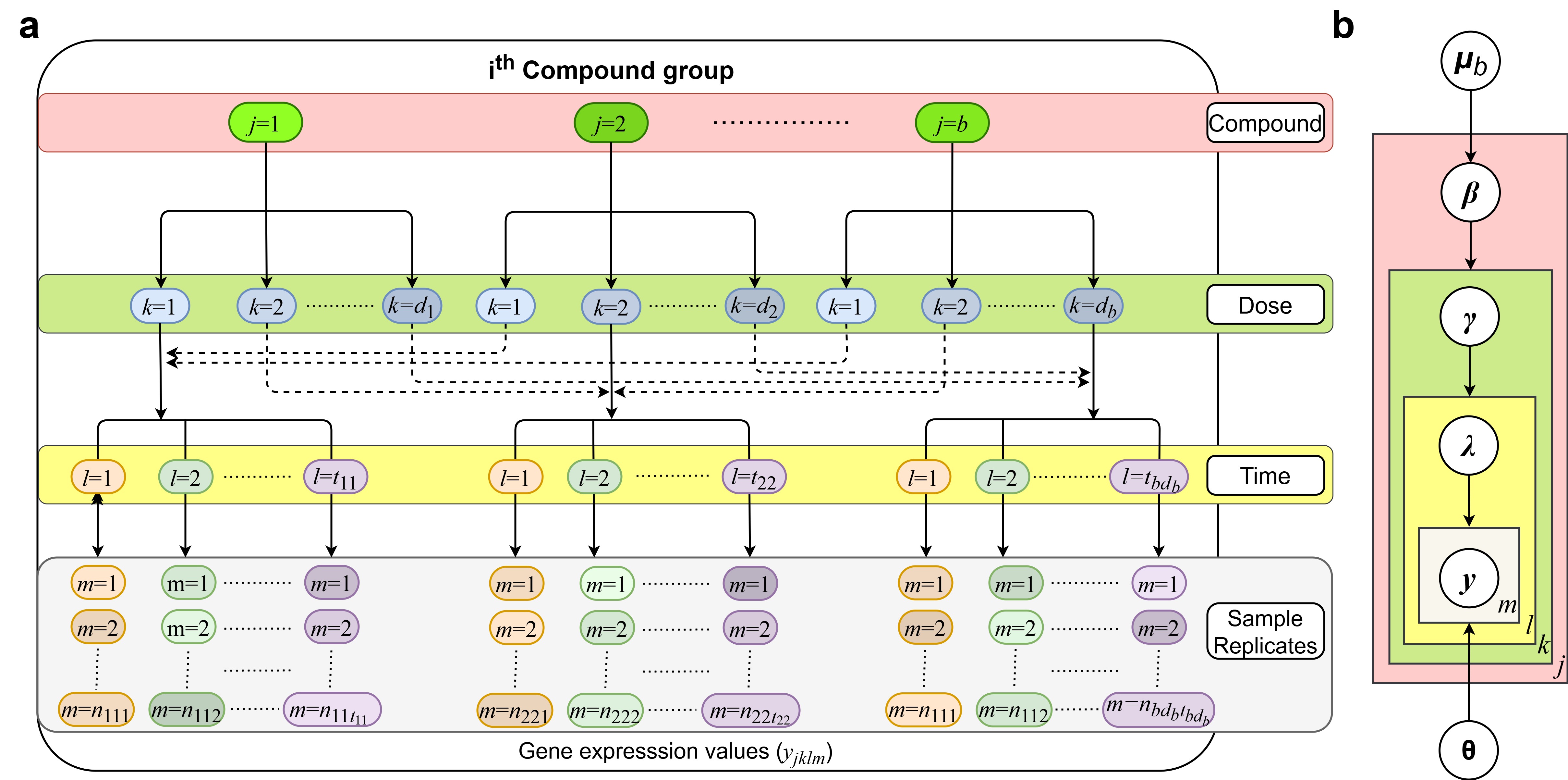


**Supplementary Fig. S1 Schematic overview of the hierarchical structure and the modeling workflow.** (**a**) A typical hierarchical data structure in toxicogenomics, where samples are treated with varying doses of compounds, then isolated at different time points, followed by imaging to obtain gene expression data. Conventional data pooling methods, which only consider sample replicates (light gray box), overlook the hierarchical structure (compound → dose → time point). The color intensity within each shape represents the magnitude of the effect or values. (**b**) The graphical model for the proposed hierarchical linear model used to analyze this time-and-dose-dependent toxicogenomics data (see Methods for details).





**Supplementary Fig. S2 Power comparison between ToxAssay, ToxicoDB, and Toxygates via simulations under unbalanced design.** Gene expression data were simulated for 10,000 genes, with 10% exhibiting a true effect based on various combinations of *r* and δ. The number of compounds in the targeted toxic ($b_{1}$) and non-toxic ($b_{2}$) groups was set randomly such that $b_{1}+b_{2}=r$ and $b_{1}\neq b_{2}$. The power of ToxAssay, ToxicoDB, and Toxygates was evaluated under four conditions: ρ = 0 (**a**-**c**), ρ = 0.1 (**d**-**f**), ρ = 0.5 (**g**-**i**), and ρ = 0.8 (**j**-**l**). Each scenario was assessed using 100 replicates at α = 0.05. The statistical power of each framework is visualized using LOESS smoothing. Contour lines indicate combinations of *r* and δ that yield equivalent statistical power, with power values quantitatively expressed. A color gradient highlights regions of varying statistical power, with low-power regions in purple and high-power regions in yellow. The x-axis represents the total number of compounds (*r*), while the y-axis represents the effect size (δ), and ρ indicates the level of data dependency. Notably, ToxAssay demonstrated increased power as ρ increased.


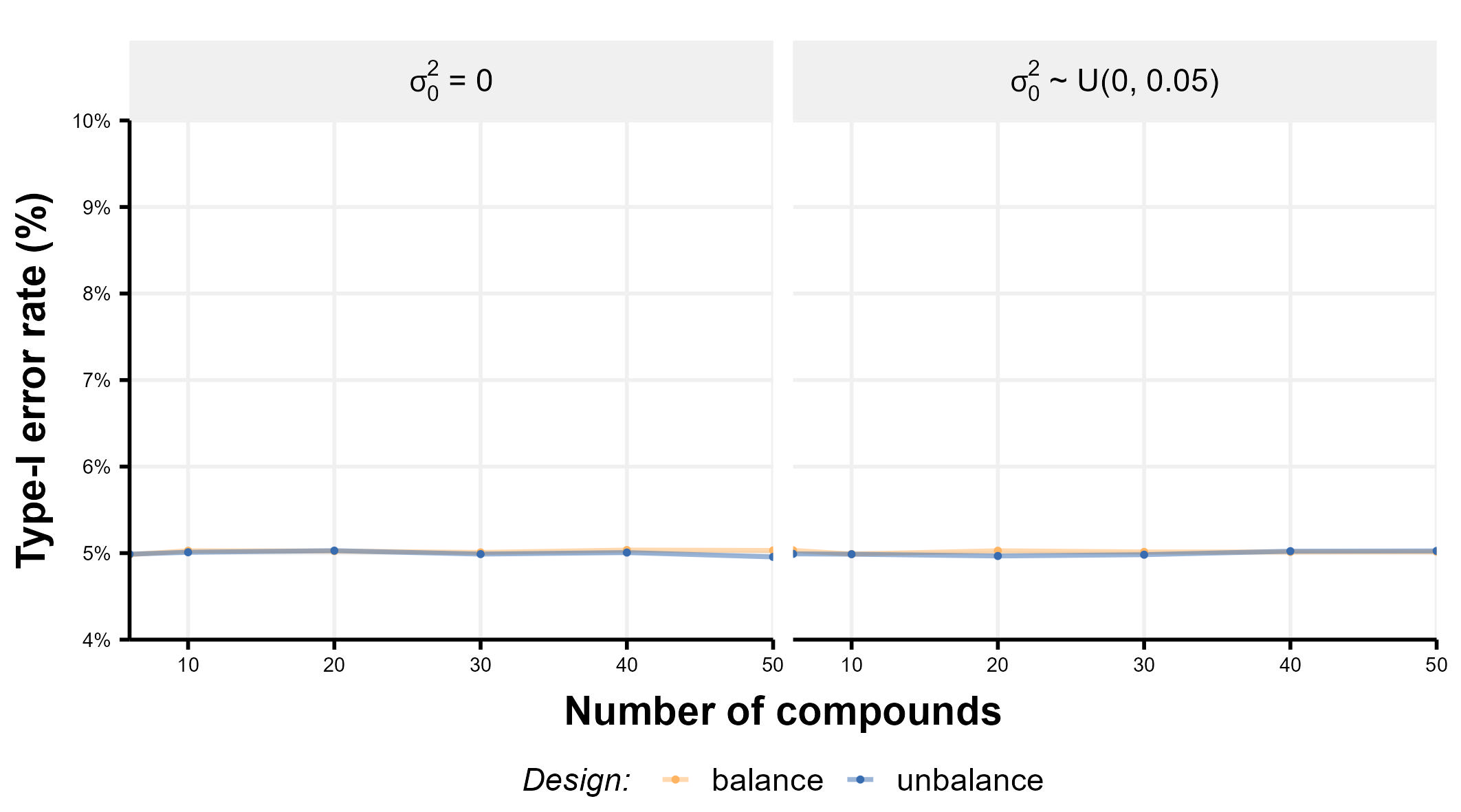


**Supplementary Fig. S3 Type-I error rate (α) of ToxAssay** **under balanced and unbalanced design.** To compare the type-I error rate (α) of ToxAssay under balanced and unbalanced designs, we simulated gene expression data for 10,000 genes. The parameter grid included the number of compounds $r\in\{6,10,20,30,40,50\}$, with $\sigma_{0}^{2}=0$ (absence of variation in the intercept) and $\sigma_{0}^{2}\sim U(0, 0.05)$ (presence of small variation in the intercepts). The x-axis is the number of compounds (*r*) and the y-axis is the type-I error rates (%). A total of 1,000 replicates were performed for each scenario, and the mean values are reported.


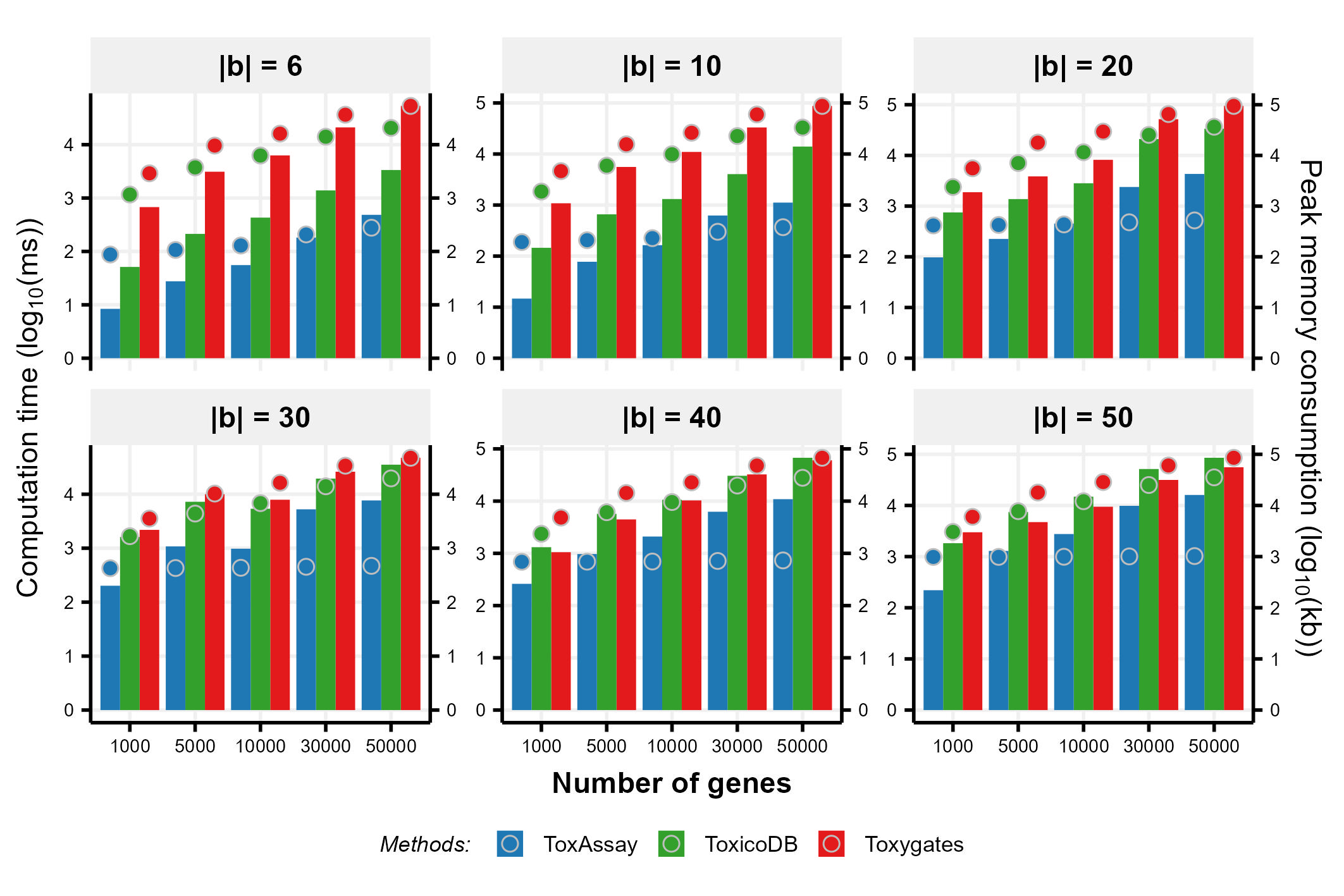


**Supplementary Fig. S4: The computational efficiency of ToxAssay, ToxicoDB, and Toxygates.** Scenarios in each configuration were replicated 100 times, with mean performance results reported using a simulated data (10, 20, 30 and 50 thousand genes; 6, 10, 20 30, 40 and 20 compounds) The bar plots (aligned with the left y-axis) represent computational time in log_10_ microseconds, while the dot plots (aligned with the right y-axis) indicate peak memory consumption in log_10_ kilobytes, across varying numbers of genes and compounds. The computational efficiency of ToxAssay, ToxicoDB, and Toxygates is depicted in blue, green, and red, respectively.


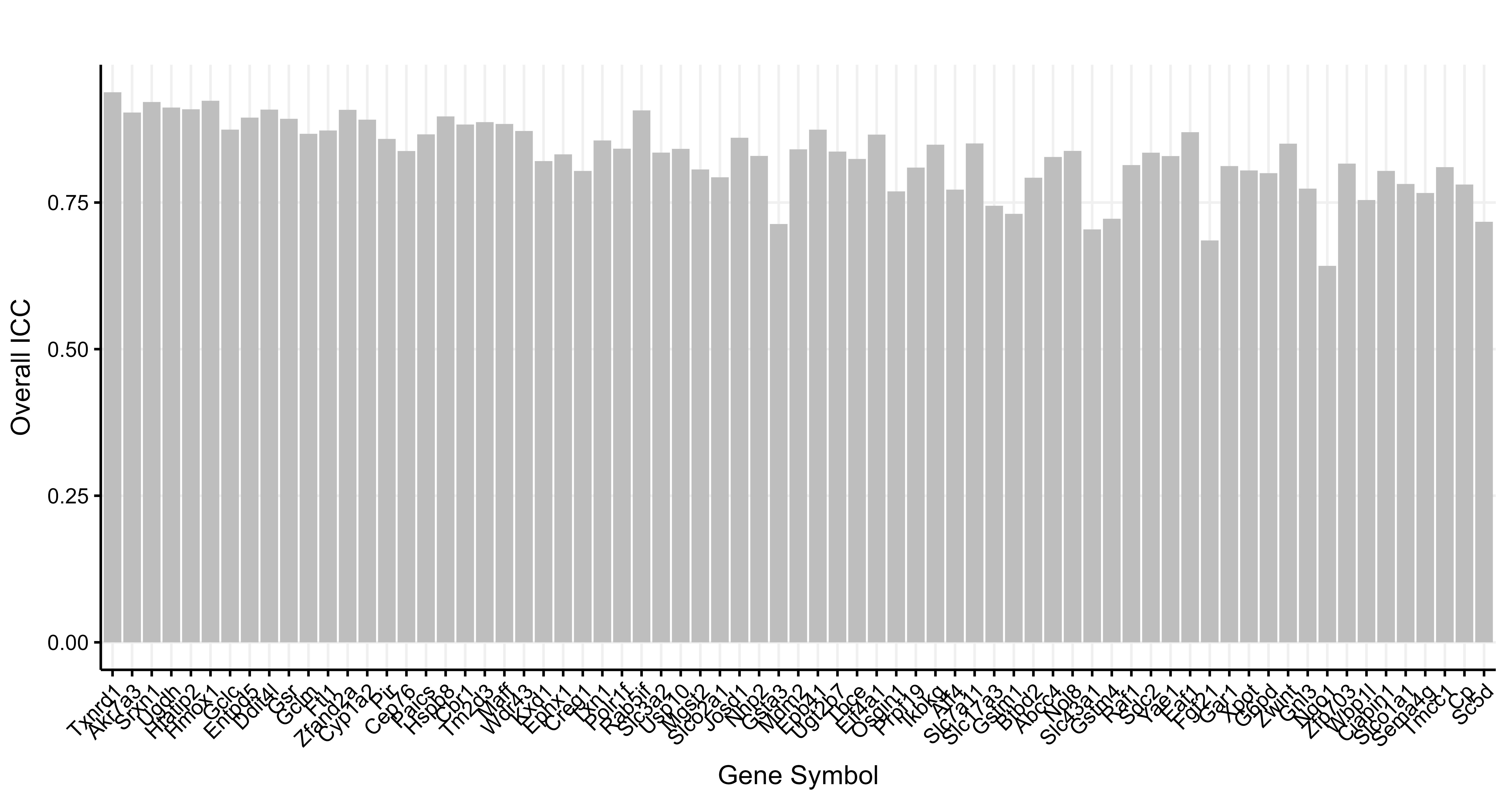


**Supplementary Fig. S5: Overall ICC across the 71 identified DEGs.** The genes are ordered based on the significance of the group mean difference between GDPCs and GDNCs. The intercept variances in the hierarchical model for determining the overall ICC were estimated using the **lme4** R package (Bates, et al., 2015).


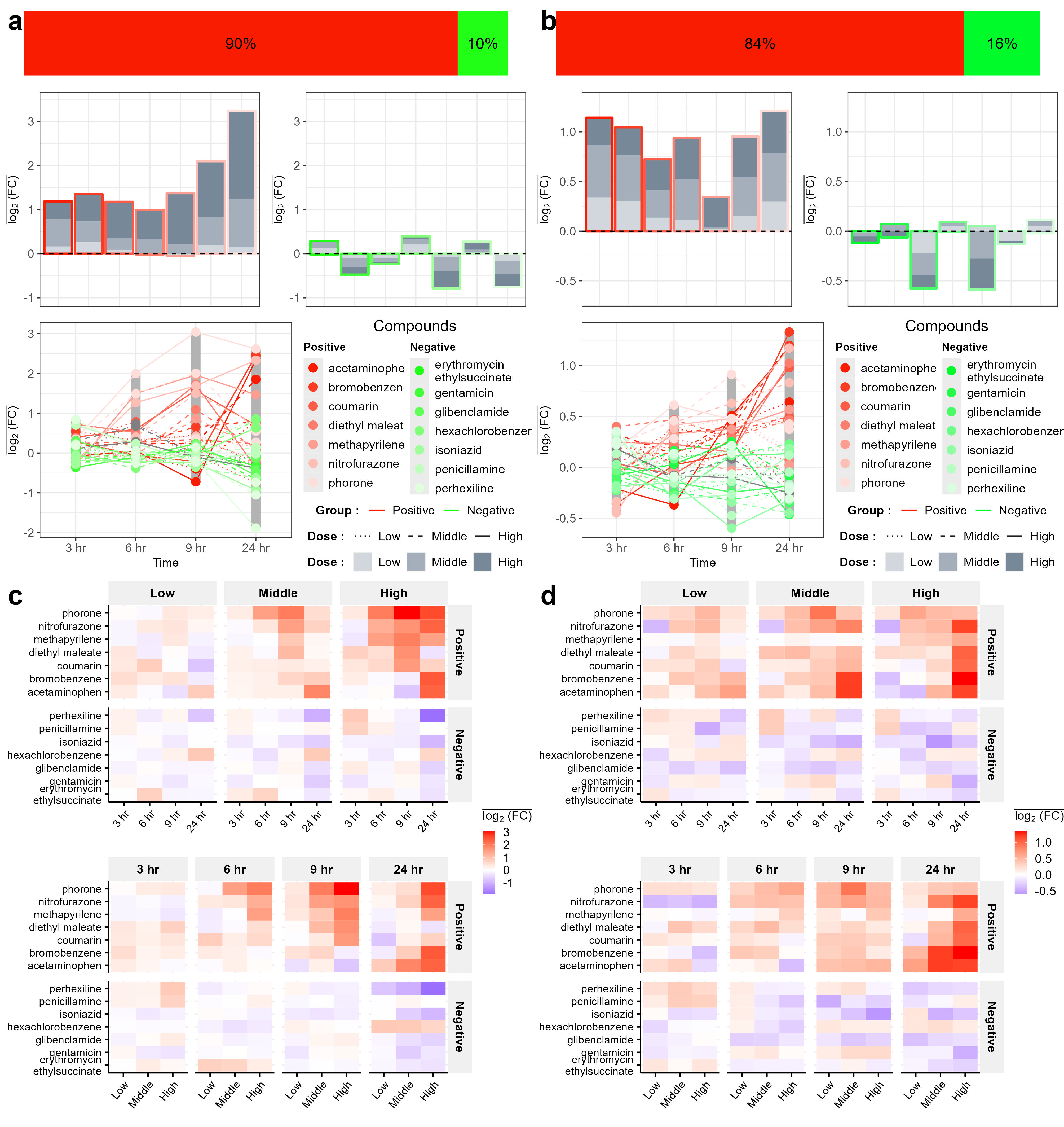


**Supplementary Fig. S6: Gene expression patterns of well-known DEGs (*G6pd* and *Mgst2*) not identified in previous studies.** (**a**) The GDPCs regulated approximately 90% of the overall expression of *G6pd*, in contrast to the minimal expression regulated by the GDNCs. *G6pd* exhibited higher average log₂(FC) in the GDPCs group across compounds, doses, and time points compared to GDNCs. **(b)** The GDPCs regulated approximately 84% of the overall expression of *Mgst2*. *Mgst2* also exhibited higher average log₂(FC) in the GDPCs group across compounds, doses, and time points compared to GDNCs. **(c)** Heatmap of *G6pd* expression clearly distinguishes GDPCs and GDNCs groups at different doses (top) and time points (bottom). (**d**) Similarly, heatmap of *Mgst2* expression clearly distinguishes GDPCs and GDNCs groups at different doses (top) and time points (bottom).


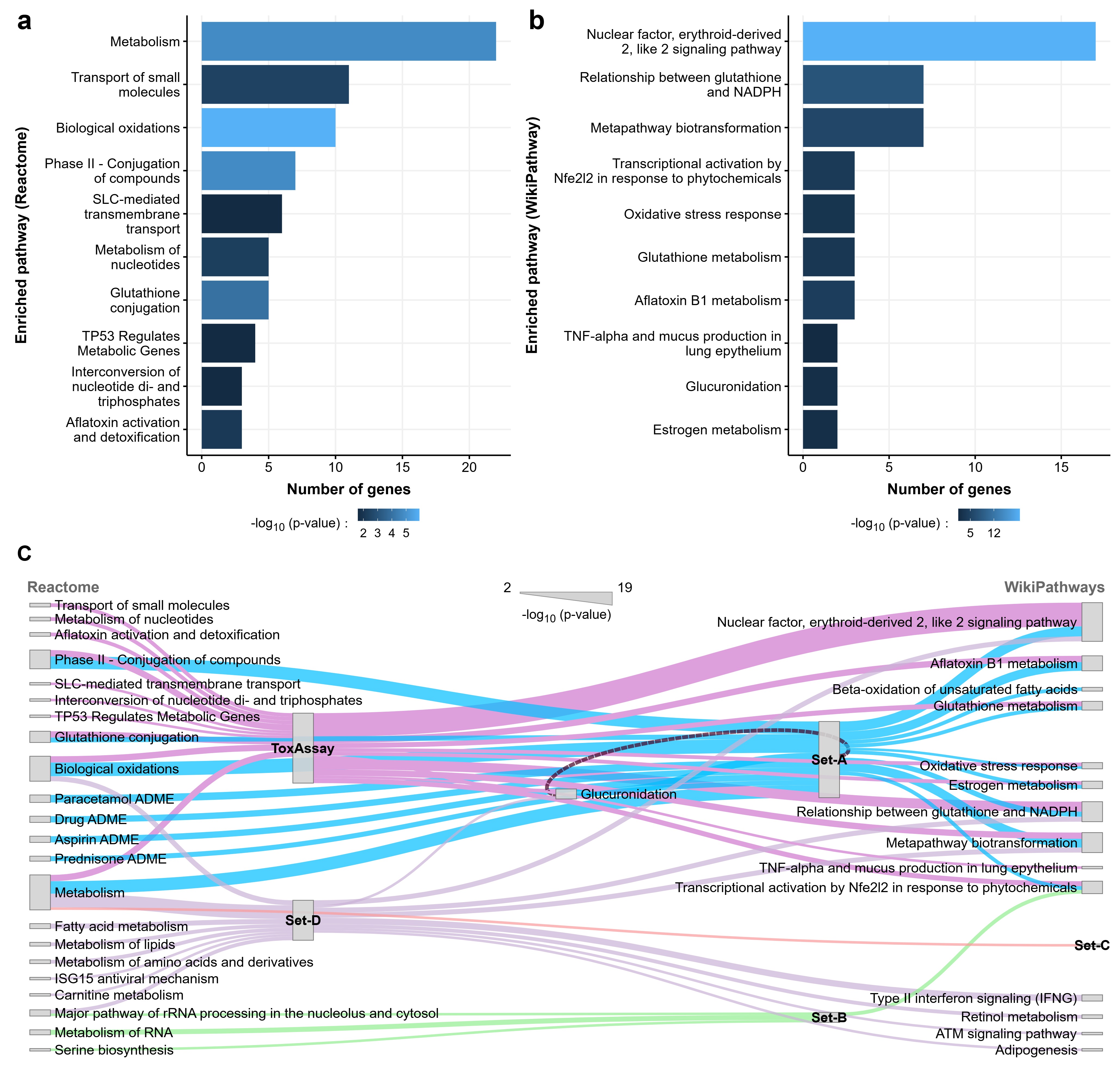


**Supplementary Fig. S7: Pathway enrichment in Reactome and Wikipathways databases.** In the top 10 pathways, 26 DEGs were significantly enriched (Q < 0.01) in Reactome pathways (**a**), and 21 DEGs were significantly enriched (Q < 0.01) in WikiPathways (**b**). The bar size representing each pathway term corresponds to the number of enriched genes. **(c)** Reactome and Wikipathways terms enriched by ToxAssay were more relevant to glutathione depletion than those identified in previous studies. The thickness of the lines reflects the -log₁₀ p-values of the pathway terms.


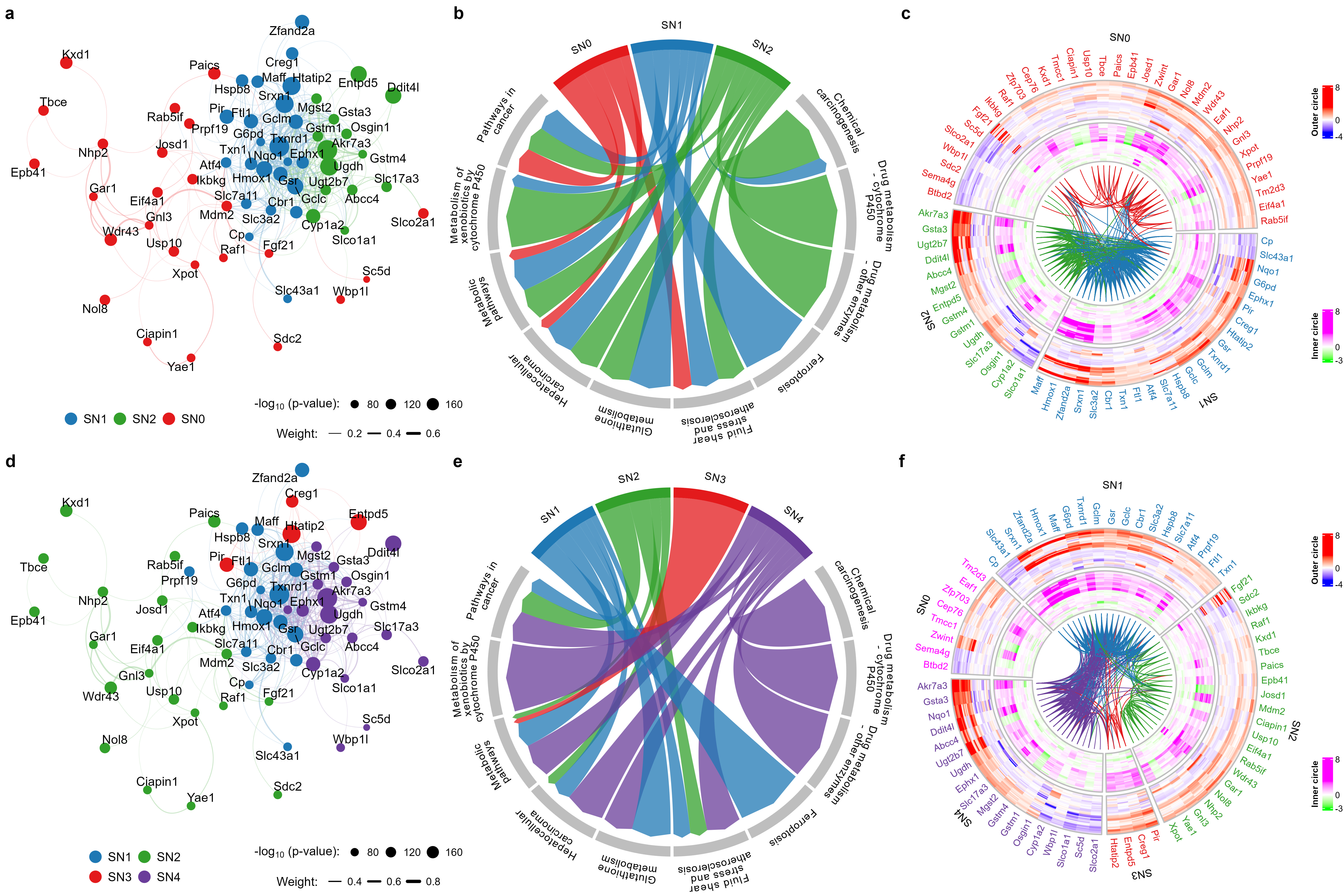


**Supplementary Fig. S8 Densest PPI subnetworks highlighting genes involved in glutathione depletion.** (**a**) The PPI network of differentially expressed genes (DEGs) revealed two distinct subnetworks (SN1, SN2) and a miscellaneous subnetwork (SN0), identified by the Walktrap algorithm. SN1 and SN2 represent the densest subnetworks. (**b**) Both SN1 and SN2 exhibited extensive pathway enrichments in KEGG. (**c**) The gene expression patterns in these subnetworks effectively distinguish GDPCs from GDNCs. The inner heatmap displays average log₂(FC) data at 24-hour high-dose levels, while the outer heatmap represents average log₂(FC) data across all time points at high-dose levels. (**d**) Similarly, the PPI network identified four distinct clusters (SN1 to SN4) using the Fastgreedy algorithm, with SN1 and SN4 being the densest subnetworks. (**e**) These subnetworks also demonstrated extensive KEGG pathway enrichments. (**f**) Gene expression patterns of genes involved in these subnetworks clearly differentiate GDPCs from GDNCs.


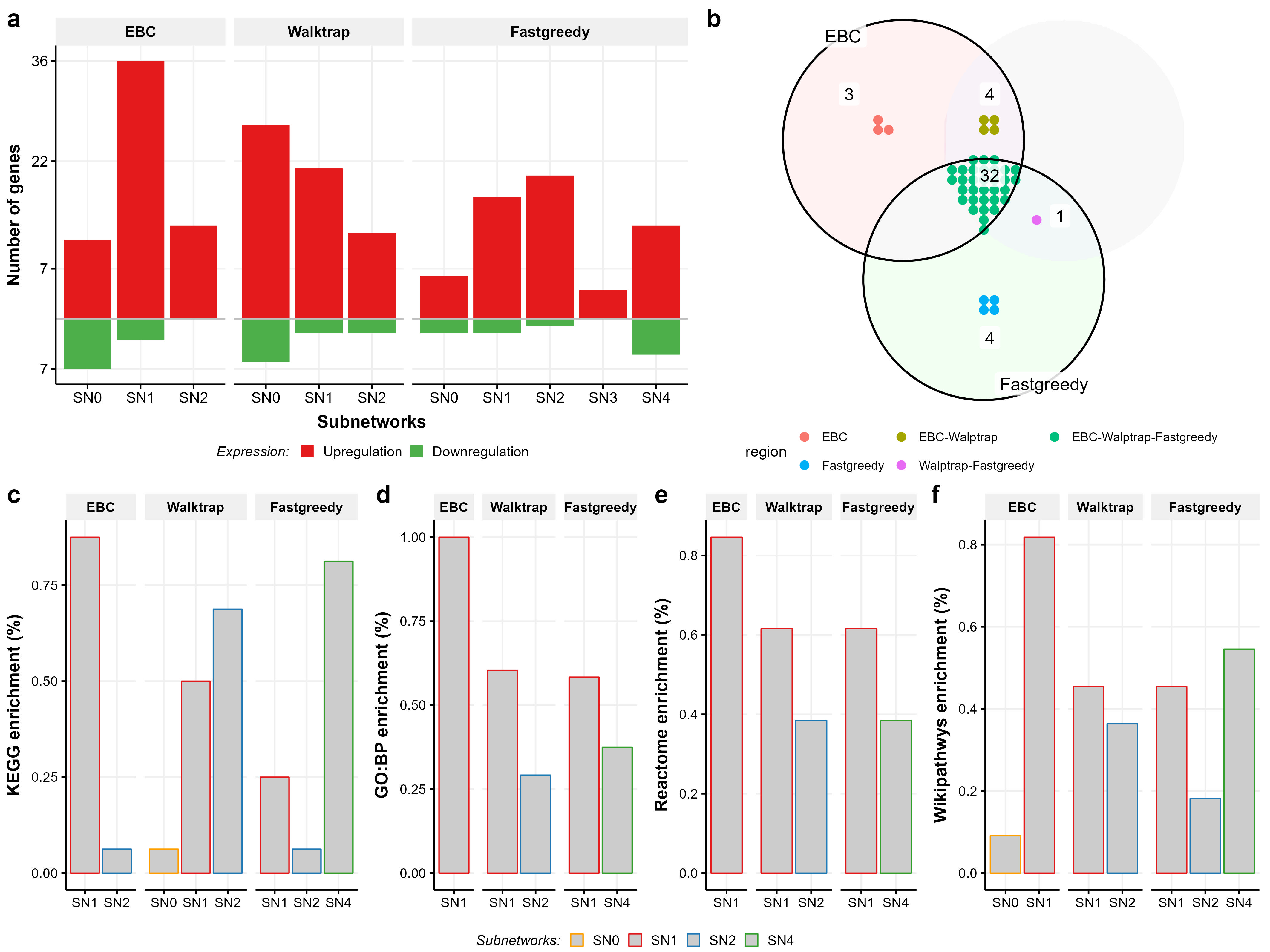


**Supplementary Fig. S9 Functional relevance of key subnetworks identified by the EBC, Walktrap, and Fastgreedy algorithms.** (**a**) Bar graph showing the number of upregulated (red) and downregulated (green) genes within subnetworks identified by the three algorithms: EBC, Walktrap, and Fastgreedy. (**b**) Venn diagram displaying the overlap and uniqueness of genes from the key subnetworks: EBC (SN1), Walktrap (SN1 and SN2), and Fastgreedy (SN1 and SN4), revealing that the genes in the key subnetworks are largely common. (**c**) Bar graphs showing pathway enrichment percentages for the key subnetworks, with EBC, Walktrap, and Fastgreedy algorithms enriching 50%-88% of significant KEGG pathways. (**d**) Pathway enrichment for GO biological process pathways, ranging from 58%-100%. (**e**) Pathway enrichment for Reactome pathways, ranging from 62%-85%. (**f**) Pathway enrichment for Wikipathways, ranging from 45%-82%. These results highlight the functional consistency of subnetworks identified by the three algorithms across multiple pathway databases.


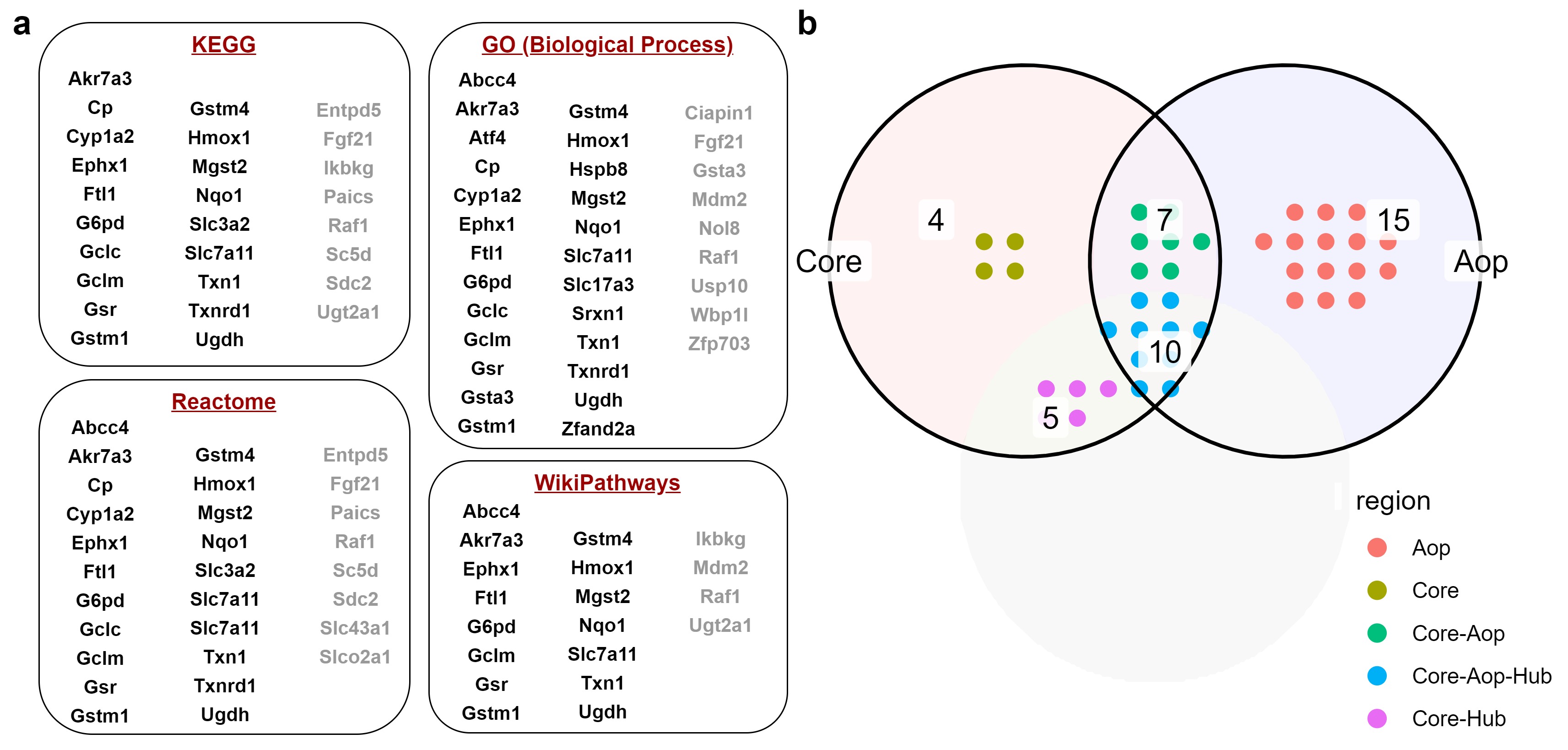


**Supplementary Fig. S10: Biological relevance of core DEGs.** (**a**) List of DEGs enriched in the top 10 pathways across multiple pathway databases (Q < 0.01). Core DEGs are highlighted in black. In the top 10 significant pathways, core DEGs account for 70.3% (19/27) in KEGG, 73.5% (25/34) in GO biological process, 72.4% (21/29) in Reactome, and 78.9% (15/19) in WikiPathways. (**b**) Venn diagram showing the overlap between core DEGs, hub genes in the PPI network, and genes significantly associated with AOPs. Of the core DEGs, 84.6% (22/26) overlap with both hub genes and AOP-related genes, with a 100% overlap with PPI hub genes. This overrepresentation of core DEGs underscores their biological relevance in glutathione depletion.


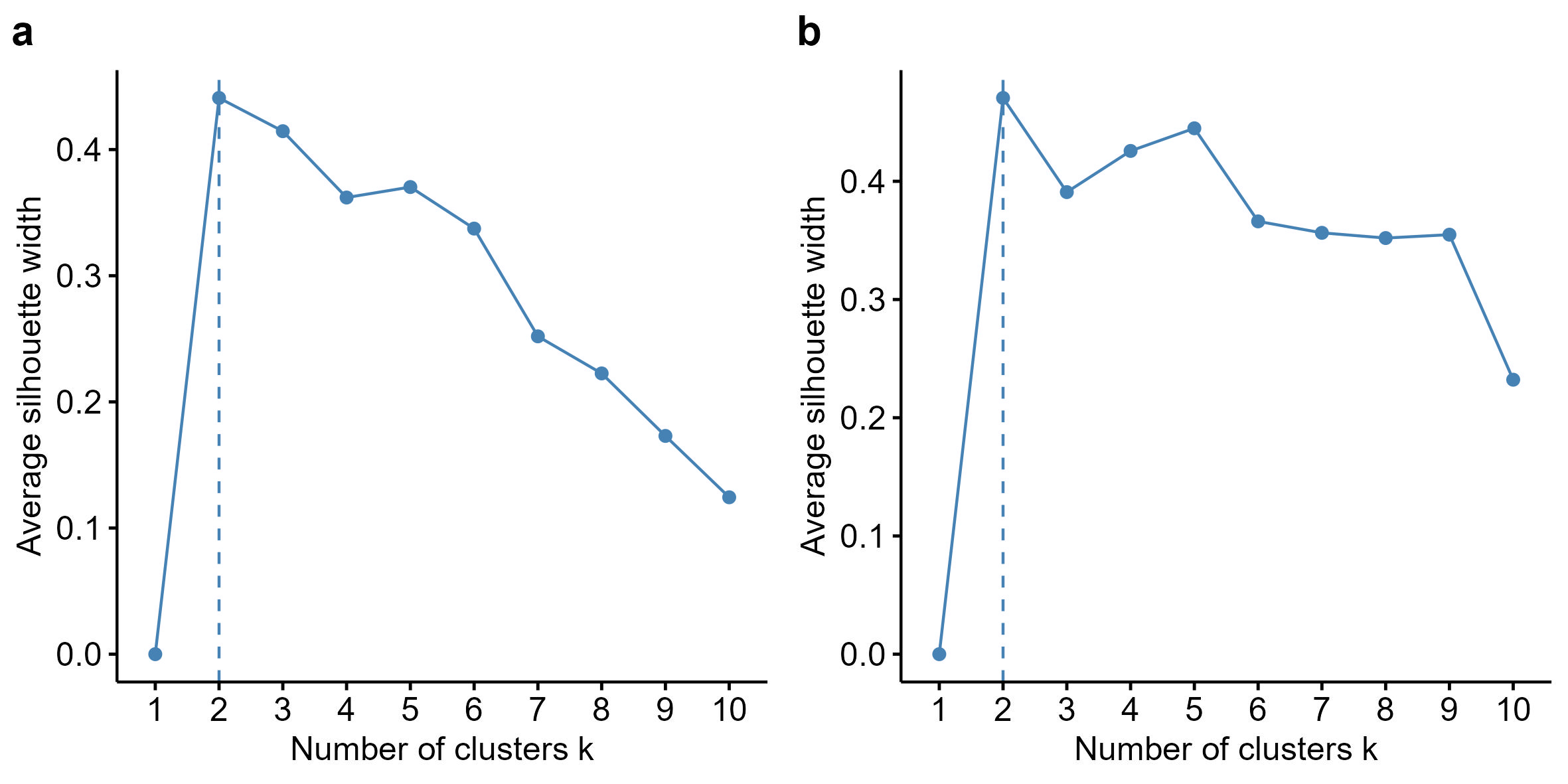


**Supplementary Fig. S11 Optimal number of clusters in different datasets.** (**a**) Hierarchical clustering of the 26 core DEGs based on average log_2_(FC) expression of high-dose samples at the 24-hour time point reveals two optimal clusters. (**b**) Clustering of data across all time points similarly identifies two optimal clusters.


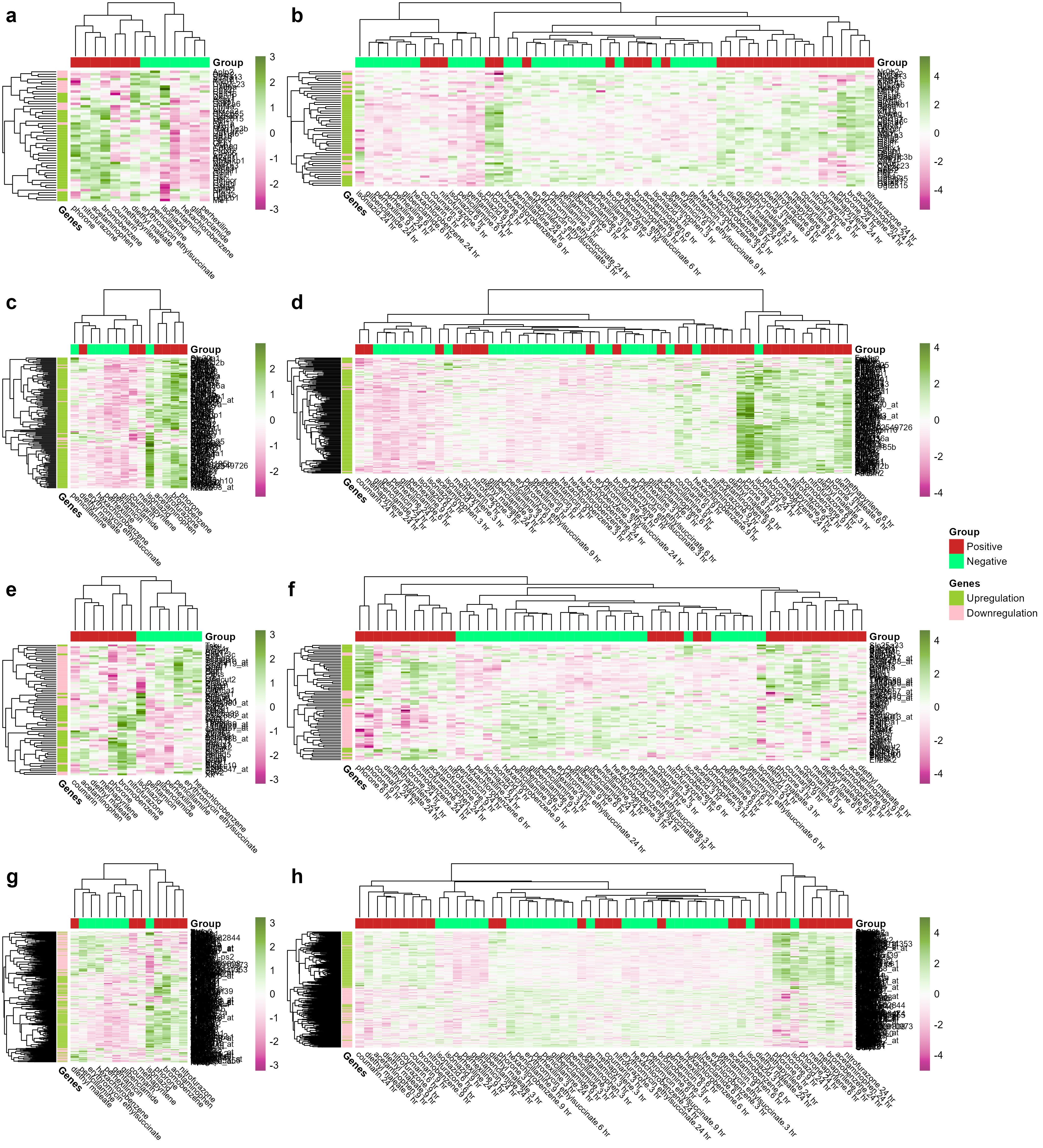


**Supplementary Fig. S12: Performance of clustering GDPCs and GDNCs using previously identified gene sets.** Hierarchical clustering based on average log₂(FC) for gene Set-A shows poor separation between high-dose GDPCs and GDNCs at the 24-hour time point (**a**) and across all time points (**b**). Similarly, hierarchical clustering using Set-B (**c**, **d**), Set-C (**e**, **f**), and Set-D (**g**, **h**) also fails to distinguish GDPCs from GDNCs at both the 24-hour time point and across all time points. Despite using different gene sets, no clear separation between GDPC and GDNC samples is observed.


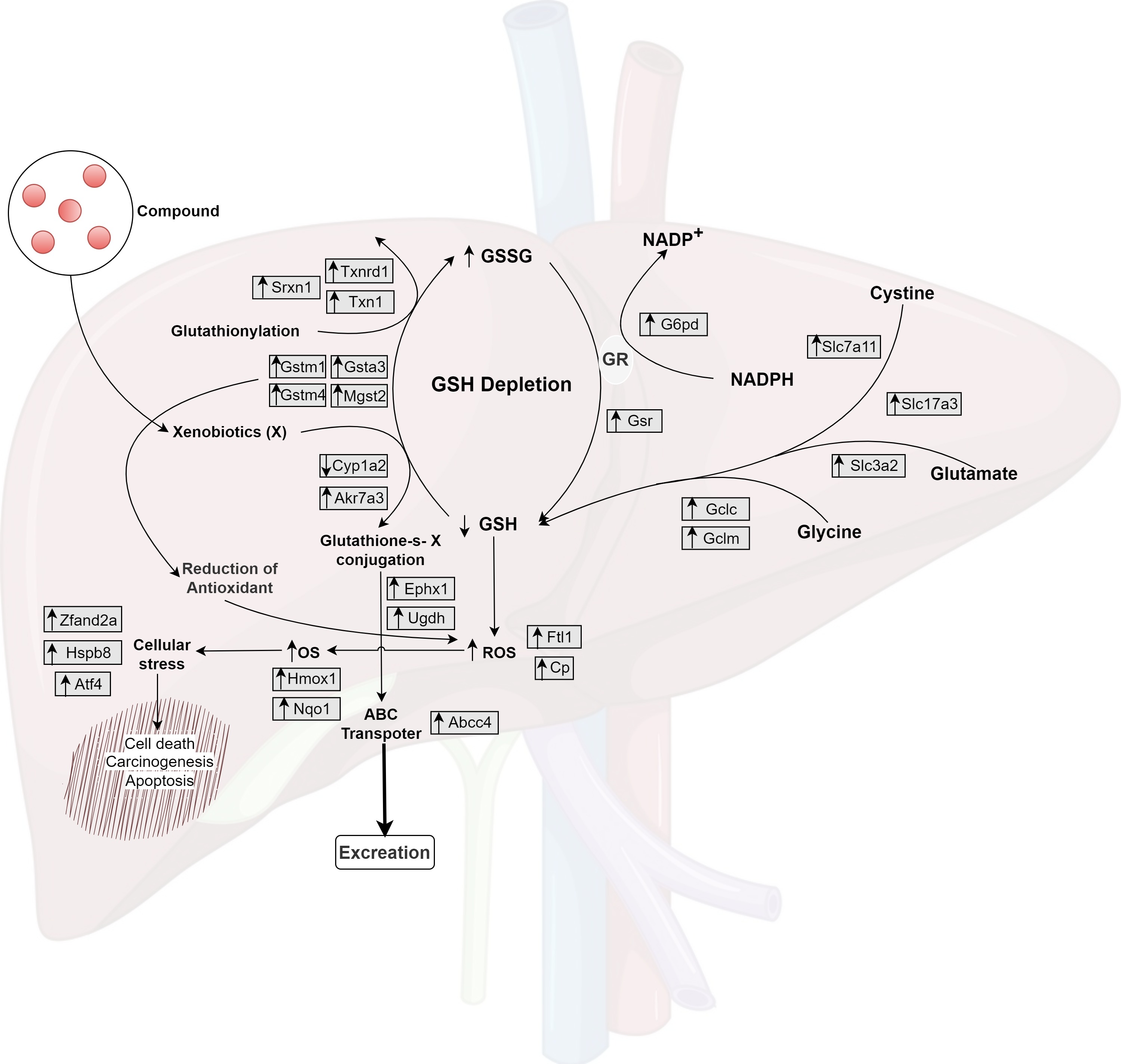


**Supplementary Fig. S13 Schematic representation of the hypothesized glutathione depletion mechanism based on 26 core DEGs**. The diagram shows the relationships between various measured parameters involved in glutathione depletion, including NADPH, NADP⁺, GR, GSH, GSSG, ROS, and OS. NADPH, reduced nicotinamide adenine dinucleotide phosphate; NADP+ oxidized nicotinamide adenine dinucleotide phosphate; GR, glutathione reductase; GSH, reduced glutathione; GSSG, oxidized glutathione; ROS, reactive oxygen species; OS, oxidative stress. Gray boxes denote the genes associated with these processes, with arrows indicating an increase in gene expression (upward arrow) or a decrease (downward arrow) under glutathione depletion conditions.
